# Flavivirus Nonstructural Protein NS5 Dysregulates HSP90 to Broadly Inhibit JAK/STAT Signaling

**DOI:** 10.3390/cells9040899

**Published:** 2020-04-07

**Authors:** Justin A. Roby, Katharina Esser-Nobis, Elyse C. Dewey-Verstelle, Marian R. Fairgrieve, Johannes Schwerk, Amy Y. Lu, Frank W. Soveg, Emily A. Hemann, Lauren D. Hatfield, Brian C. Keller, Alexander Shapiro, Adriana Forero, Jennifer E. Stencel-Baerenwald, Ram Savan, Michael Gale

**Affiliations:** 1 Center for Innate Immunity and Immune Disease, Department of Immunology, University of Washington School of Medicine, Seattle, WA 98109, USA; ken10@uw.edu (K.E.-N.); edewey@uw.edu (E.C.D.-V.); marianfairgrieve@gmail.com (M.R.F.); jschwerk@uw.edu (J.S.); amyyhlu@uw.edu (A.Y.L.); fsoveg@uw.edu (F.W.S.); eahemann@uw.edu (E.A.H.); ldaarreberg@gmail.com (L.D.H.); forera@uw.edu (A.F.); Jen.Baerenwald@gmail.com (J.E.S.-B.); savanram@uw.edu (R.S.); 2 Departments of Global Health and Microbiology, University of Washington, Seattle, WA 98195, USA; 3 Division of Pulmonary, Critical Care & Sleep Medicine, The Ohio State University College of Medicine, Columbus, OH 43210, USA; Brian.Keller2@osumc.edu; 4 Shorewood High School, Shoreline, WA 98133, USA; ags245@cornell.edu

**Keywords:** flavivirus, JAK/STAT, cytokine, West Nile virus, *Zika virus*, HSP90, NS5, virus–host interactions, anti-viral signaling, immune response

## Abstract

Pathogenic flaviviruses antagonize host cell Janus kinase/signal transducer and activator of transcription (JAK/STAT) signaling downstream of interferons α/β. Here, we show that flaviviruses inhibit JAK/STAT signaling induced by a wide range of cytokines beyond interferon, including interleukins. This broad inhibition was mapped to viral nonstructural protein 5 (NS5) binding to cellular heat shock protein 90 (HSP90), resulting in reduced Janus kinase–HSP90 interaction and thus destabilization of unchaperoned JAKs (and other kinase clients) of HSP90 during infection by *Zika virus*, West Nile virus, and Japanese encephalitis virus. Our studies implicate viral dysregulation of HSP90 and the JAK/STAT pathway as a critical determinant of cytokine signaling control during flavivirus infection.

## 1. Introduction

The genus *Flavivirus* comprises several important human pathogens including West Nile virus (WNV), dengue virus (DENV), Japanese encephalitis virus (JEV), and *Zika virus* (ZIKV). These (+)-sense, single-stranded RNA viruses are transmitted to humans by mosquito bite and represent (re)emerging viruses. Roughly half of the world’s population is at risk of DENV infection, causing ≈390 million infections and 21,000 deaths per year [1]. Since emergence in the USA, WNV has spread to all contiguous states causing ≈2340 deaths (The US Centers for Disease Control and Prevention (CDC) records as of August 2019), and is a model example of emerging infectious disease [2]. Recently, ZIKV emerged with outbreaks in Oceania and Latin America [3,4]. ZIKV infection causes symptoms ranging from flu-like illness to Gullain–Barré syndrome [4]. ZIKV can undergo maternal–fetal transmission, causing congenital Zika syndrome (CZS) marked by microencephaly and post-natal cognitive disorders [5,6,7,8,9]. ZIKV is of global concern but the virus–host interactions of ZIKV infection linked with CZS are not fully understood. Defining the processes in which flaviviruses employ to evade and antagonize host immune responses is paramount to informing vaccine and therapeutic strategies to combat infection. No approved vaccines or specific antivirals are currently available to prevent or treat human infections with DENV, WNV, or ZIKV.

The immune response to flavivirus infection initiates upon viral recognition by host cell pattern recognition receptors (PRRs) [10,11]. PRR signaling in the infected cell leads to activation of interferon regulatory factor (IRF) 3 and induction of interferons (IFNs) α, β, and λ. IFN secretion and binding to the cognate receptor then triggers the Janus kinase/signal transducer and activator of transcription (JAK/STAT) signaling cascade for rapid induction of IFN-stimulated genes (ISGs). Janus kinases JAK1, JAK2, and Tyk2 phosphorylate STAT1 and STAT2 on specific tyrosine residues (pY) in response to IFN/receptor interaction. pY-STATs heterodimerize and move to the nucleus to bind target promoters inducing ISG expression [12]. ISG products establish an antiviral state in responding cells to restrict virus replication and spread. The innate immune response is essential for control of flavivirus infection [13]. Flavivirus antagonism of IFN-responsive JAK/STAT signaling is a feature associated with virulence [14,15,16,17,18,19]. Flavivirus nonstructural protein 5 (NS5) has been identified as the primary mediator of IFN signaling inhibition [14]. Roles in JAK/STAT antagonism have also been attributed to other NS proteins and subgenomic flavivirus RNA [16,20,21,22,23].

In addition to IFN signaling, the JAK/STAT pathway is utilized by several distinct cytokines that activate various Janus kinases (JAKs; including JAK1, JAK2, JAK3, and Tyk2) upon receptor interaction [24,25]. However, flaviviral inhibition of JAK/STAT signaling by cytokines other than IFN remains unexplored. We examined flavivirus regulation of JAK/STAT signaling induced by IFNs β, γ, and λ3 as well as inflammatory (IL6), and also immune-regulatory (IL4, IL10) cytokines that utilize this pathway. We revealed the fact that flaviviruses broadly inhibit JAK/STAT signaling across STAT1-6 to disrupt responses to IFNs, as well as pro-inflammatory and immune-regulatory cytokines. Mechanistically, this broad antagonism of JAK/STAT signaling was mediated by flavivirus NS5 binding to host heat shock protein 90 (HSP90), leading to dysregulation of chaperone stabilization (and eventual subsequent degradation) of JAKs, as well as abrogation of cytokine-induced pY-STAT. Unlike previous reports of flavivirus interaction with other heat shock proteins [26,27,28], we showed that flaviviruses including ZIKV do not usurp chaperone activity to promote viral protein function and genome replication. Rather, flaviviruses appear to specifically target and disrupt HSP90 chaperone activity to suppress host JAK/STAT signaling, thus controlling the actions of a broad range of immunoregulatory and antiviral cytokines. Importantly, this broad-acting innate immune evasion strategy has never before been identified.

Thus, HSP90 is a key virus-targeted host factor, wherein interaction with viral NS5 prevents its interaction with client kinases, resulting in suppression of JAK/STAT-dependent cytokine signaling. NS5 targeting of HSP90 is thus the mechanism that underlies the disruption of broad cytokine signaling by flaviviruses.

## 2. Materials and Methods

### 2.1. Contact for Reagent and Resource Sharing

Further information and requests for resources and reagents should be directed to and will be fulfilled by the Lead Contact, Prof. Michael Gale Jr. (mgale@uw.edu).

### 2.2. Cell Lines

A549, Vero, HEK-293T, Huh-7 replicon-cured, PH5CH8 cells with interferon alpha receptor 1 knockout (IFNAR1^−/−^), and murine embryonic fibroblast (MEF) cells were routinely cultured in complete Dulbecco’s modified Eagle’s medium (cDMEM; Corning, USA) supplemented with 10% (v/v) heat-inactivated fetal bovine serum (FBS; Hyclone, Logan, UT, USA), 2 mM L-glutamine, 1 mM sodium pyruvate, 10 mM 4-(2-hydroxyethyl)-1-piperazineethanesulfonic acid (HEPES), 1X antibiotic/antimycotic (Corning), and 1X non-essential amino acids (Corning, Corning, NY, USA). U251-MG cells were cultured in DMEM supplemented with 10% (v/v) FBS (Hyclone, Logan, UT, USA), 2 mM L-glutamine, 1.14 mM sodium pyruvate, 1X antibiotic/antimycotic (Corning), and 1X non-essential amino acids (Corning). THP-1 cells were cultured in complete Roswell Park Memorial Institute medium (cRPMI) supplemented with 10% (v/v) heat-inactivated fetal bovine serum (FBS; Hyclone, Logan, UT, USA), 2 mM L-glutamine, 1 mM sodium pyruvate, 10 mM HEPES, 1X antibiotic/antimycotic (Corning), and 1X non-essential amino acids (Corning). Prior to use in experiments, THP-1 cells were differentiated into macrophage-like cells via overnight stimulation with 40 nM phorbol myristate acetate (PMA) in cRPMI. Huh-7 WNrep cells were cultured in cDMEM supplemented with 1 mg/mL G418 to select for maintainence of the replicon. During cytokine stimulation, replicon cells were switched to cDMEM without G418 to standardize conditions between these and replicon-cured cells. All cell lines were confirmed as being free of mycoplasma contamination.

### 2.3. Virus Strains

Flaviviruses WNV-TX, WNV-MAD, ZIKV MR766, ZIKV FSS13025, ZIKV Fortaleza, JEV Nakayama, DENV-2 New Guinea C, and DENV-4 H241 were grown and titred on Vero cell monolayers in cDMEM. All infections were initiated using a low volume inoculum of virus in complete media (media dependent on cell type) incubated at 37 °C for 2 h with rocking. Inoculum was subsequently removed and replaced with fresh complete media. The encephalomyocarditis virus (EMCV) strain Mengo infectious clone was a kind gift from Ann C. Palmenberg (UW-Madison). Virus stocks were grown and titred on Vero cell monolayers in cDMEM. Sendai virus (SeV) Cantell strain was sourced from Charles River Laboratories (USA). All virus stocks were confirmed to be free of mycoplasma contamination.

### 2.4. Transcript Analysis by qRT-PCR

Cells were lysed and homogenized using QIAshredders (QIAGEN, Hilden, Germany), and total cellular RNA was isolated using a RNeasy Kit (QIAGEN) with DNase I (QIAGEN) digestion on column. Purified RNA was converted to complimentary DNA (cDNA) using the iScript cDNA Synthesis Kit (Bio-Rad, Hercules, CA, USA). The resulting cDNA was analyzed by qRT-PCR using SYBR Green Master Mix (ThermoFisher, Waltham, MA, USA) and gene-specific primers on the ABI 7500 Real-Time PCR System.

### 2.5. Protein Analysis by Western Blot

Cells were washed 1 x with phosphate buffered saline (PBS) and then lysed in radioimmunoprecipitation assay (RIPA) buffer (50 mM Tris pH 7.4, 150 mM NaCl, 1% (v/v) Triton X-100, 0.5% (w/v) sodium deoxycholate, 1:100 phosphatase inhibitor cocktail (VWR, Radnor, PA, USA), 1:100 protease inhibitor cocktail (Sigma, St. Louis, MO, USA), 250 nM okadaic acid) on ice. Lysates were scraped into microfuge tubes and immediately frozen at −80 °C. Lysates were subsequently thawed on ice for >30 min, then sonicated in a water bath ice slurry for 3 × 30 s bursts on a high setting with 2 × 20 s pauses between. Nuclei and other cellular debris were cleared from lysate via centrifugation at 14,000 rpm for 15 min at 4 °C. Protein content was quantified using a Bio-Rad Protein Assay (Bio-Rad), and samples were stored at −80 °C until required.

Prior to loading, lysates were incubated with 4X Laemmli buffer +/− β-mercaptoethanol at 95 °C for 3 min. Samples were loaded (≈7–14 μg total protein per lane) onto 4–20% Criterion TGX gels (Bio-Rad) and electophoresed at 97 V in Western running buffer (25 mM Tris, 192 mM glycine, 0.1% (w/v) SDS). Subsequently, proteins were transferred to nitrocellulose membranes at 90 V for 1 h under submerged conditions in Western transfer buffer (25 mM Tris, 192 mM glycine, 0.01% (w/v) SDS, 20% (v/v) methanol). Membranes were blocked for 1 h at RT in Tris-buffered saline (TBS)-based Odyssey blocking buffer (LI-COR, Lincoln, NE, USA), and subsequently stained overnight with primary antibody (see key resources table) in TBS-based Licor blocking buffer at 4 °C. The following day, membranes were washed 3x with TBS plus Tween 20 (TBST), and probed with secondary antibody (either horseradish peroxidase (HRP)-, Alexa680-, or Alexa790-conjugated antibody; key resources table) in TBS-based Odyssey blocking buffer for 1 h at RT. Finally, membranes were washed 3x with TBST and 2x with TBS prior to either direct imaging on an Odyssey CLx imager (LI-COR), or band development using enhanced chemiluminescence (ECL) prime Western blotting reagent (Fisher Scientific) and detection on a ChemiDoc XRS+ system (Bio-Rad).

### 2.6. Immunofluorescence Assays

Cells seeded on coverslips or chamber slides and used for experiments were washed 1x with PBS and were fixed with 4% paraformaldehyde in TBS for 30 min at room temperature. Following this, cells were washed with filtered 1X TBS and permeabilized (for pY-STAT staining, fixation was for 10 min at −20 °C with 100% ice-cold methanol and drying cells completely, followed by a further 3 washes with TBS; for all other samples, fixed cells were permeabilized via addition of 0.1% Triton X-100 to the blocking buffer, with permeabilization occurring during the blocking step). Blocking of cells was for 30 min at RT with filtered 5% normal goat serum (NGS) in TBS. Cells were then stained for 1 h at RT or overnight at 4 °C with primary antibody in 5% NGS/TBS. Subsequently, cells were washed 3x with TBS and probed for 1 h at RT with secondary antibody plus 1:10,000 DAPI (4’,6-diamidino-2-phenylindole dihydrochloride) in 5% NGS/TBS. Finally, cells were washed 3x with TBS; briefly washed 1x with deionized water (dH_2_O) to remove excess salt; and carefully dried on the back of the coverslip, removing excess water. Coverslips were mounted onto slides using ProLong Gold mounting media and dried at least overnight at RT. Confocal immunofluorescence images were acquired on a Nikon Eclipse Ti microscope and analyzed using the NIS-Elements imaging system software (version 4.51) (Nikon Instruments, Tokyo, Japan). For some images, the red channel was modified equally via a saved look-up table (LUT) setting across all samples per experiment to aid visual clarity post-acquisition.

### 2.7. Conditioned Media Experiments

A549 cells were mock infected or infected with WNV-TX at multiplicity of infection (MOI) = 5 for 24 h. Cell-free culture media was harvested, divided into two equal volumes per condition, and half subjected to UV inactivation for 30 min in a Spectrolinker XL-1000 UV crosslinker (Spectronics Corporation, Westbury, NY, USA) at full power on ice. Normal and UV-inactivated conditioned media was then aliquoted and stored at −80 °C. Thawed conditioned media was subsequently used neat in the treatment of seeded A549 cells for 24 h prior to acute, 30 min cytokine treatment. Treated cells were lysed post-stimulation and analyzed via Western blot.

### 2.8. Generation of Recombinant DNA Constructs

Plasmids pCAGGS-HA and pCAGGS-WNV-NS4B were kindly provided by Adolfo Garcia-Sastre (Munoz-Jordan et al., 2005). Each gene from WNV strain TX02 was amplified from cDNA and cloned into the mammalian expression vector pCAGGS-HA in-frame with the 3’-HA tag. The 5’ end of amplified WNV genes included either EcoRI, NsiI, or SacI restriction sites followed by an AUG start codon while 3’ end contained KpnI or NsiI restriction sites for insertion into the multiple cloning site of pCAGGs-HA. Because the amino acid sequence of WNV strain NY99 NS4B from pCAGGS-WNV-NS4B (Munoz-Jordan et al., 2005) was identical to that of TX02 NS4B, the NY99-based construct pCAGGS-WNV-NS4B was used in these studies.

Plasmid pTwist-CMV-HSP90-HA was designed in-house and custom synthesized by Twist Bioscience (USA). The vector plasmid pTwist-CMV was created via excision of the HSP90-HA coding sequence with a Not-I/Nhe-I digestion, followed by blunting of the terminal ends via digestion with Mung Bean Nuclease (New England Biolabs, Ipswich, MA, USA), and finally blunt-end ligation. pTwist-CMV-FLAG-JAK1 was created via amplification of human JAK1 from a cDNA open reading frame (ORF) cloning vector (Sino Biological, Beijing, China) with gene-specific primers incorporating an N-terminal FLAG tag. This PCR product was then digested with Not-I/Nhe-I and ligated into the pTwist–CMV vector.

The vector pcDNA3.1(+) was purchased from Thermo Fisher Scientific (USA) and the plasmids pcDNA3.1-ZIKV(C)-FLAG and pcDNA3.1-ZIKV(NS5)-FLAG were the kind gift of Tom C. Hobman [29].

### 2.9. Transfection of Nucleic Acids

Plasmid DNA was transfected into cells using Lipofectamine 3000 (ThermoFisher) according to the manufacturer’s instructions. A549 cells seeded into 24-well plates on coverslips for immunofluorescence assays were transfected with 1 µg/well DNA, and HEK-293T cells seeded into 6-well plates for co-immunoprecipitation (co-IP) assays were transfected with 5 µg/well total DNA (when co-transfected, this amount was split equally between plasmids; i.e., 2.5 µg/well each). The ratio DNA (µg)/lipofectamine 3000 (µL)/P3000 reagent (µL)/Opti-MEM (µL) was 1:3:2:50. Cell media was freshly changed prior to transfection. Plasmid DNA and P3000 were added to half the volume Opti-MEM (ThermoFisher) and mixed by inversion; lipofectamine 3000 was added to the other half of Opti-MEM and mixed. The two solutions were combined, mixed by inversion, and incubated 15 min at RT prior to drop-wise addition to cells.

### 2.10. Co-IP of Recombinant FLAG- and HA-tagged Proteins

HEK-293T cells seeded in 6-well plates and used for experiments were washed 1x with PBS and then lysed in 250 µL/well co-IP lysis buffer + inhibitors (50 mM Tris HCl pH 7.5, 250 mM NaCl, 5 mM ethylenediaminetetraacetic acid (EDTA), 0.02% (w/v) sodium azide, 1% (v/v) NP40, 1:100 phosphatase inhibitor cocktail (VWR), 1:100 protease inhibitor cocktail (Sigma), 250 nM okadaic acid) on ice. Lysates were scraped into new microcentrifuge tubes and incubated on ice for at least 30 min. Lysates were cleared of cellular debris via centrifugation at 14,000 rpm, 15 min, 4 °C, with supernatant transferred to fresh tube on ice and quantified as above. For each co-IP, 250 µg of each sample (in a 200 µL volume—excess lysis buffer was used to make volume) was incubated overnight at 4 °C in tubes on rotator with the following conditions. *For anti-HA*: 10 µL of thoroughly vortexed anti-HA magnetic beads (Cell Signaling Technology, Danvers, MA, USA) were added to each 200 µL sample, and were rotated overnight at 4°C. *For anti-FLAG*: Pre-binding of 1 µL anti-FLAG antibody (Sigma) to each 200 µL sample was performed on rotator at 4 °C for 15 min. Subsequently, 10 µL of vortexed protein G magnetic beads (ThermoFisher) were added to each 201 µL sample, and were rotated overnight at 4 °C.

The following day samples were immunoprecipitated using a magnet. Supernatant was discarded and 500 µL co-IP lysis buffer + inhibitors was added to each tube. These were briefly inverted to mix and rotated at 4 °C for 5 min. Magnetic precipitation and washing was repeated two more times (three washes total) using 300 µL for repeat washes.

Co-IP lysis buffer + inhibitors were used to dilute 4X Laemmli sample buffer (Bio-Rad) + β-mercaptoethanol to form a 1X sample buffer. A total of 50 µL of this buffer was added per sample as a final resuspension/elution. Samples were stored at −80 °C.

When loading gels, samples were incubated 3 min at 95 °C and then placed immediately on ice. Samples were spot centrifuged >10 s to pellet beads. Samples were then analyzed by Western blot as appropriate, with 10–15 µL co-immunoprecipitate loaded per lane.

### 2.11. ReCLIP Analysis of Endogenous Proteins

The protocol was modified from [30]. A549 cells infected with flaviviruses at MOI = 5 for 24 h were washed 2x with PBS prior to crosslinking proteins for 30 min at RT with 0.5 mM dithiobis(succinimidyl propionate) (DSP) in PBS. Crosslinked cells were subsequently quenched with incubation for 10 min at RT with TBS. Cells were then washed 1x with TBS and lysed on ice for 30 min with RIPA buffer followed by scraping into microcentrifuge tubes. Lysates were briefly sonicated in an ice-slurry bath (2 × 20 s pulses on the high setting with a 30 s pause). Lysate was cleared via centrifugation at 14,000 rpm for 15 min at 4 °C.

Lysate was pre-cleared by incubating 600 µg of total protein with 15 µL Protein G Dynabeads (ThermoFisher) in 1300 µL final volume for 30 min at 4 °C with rotation. For each IP, 150 µg of this cleared lysate was incubated with appropriate antibodies (2 µg mouse immunoglobulin G 2a (IgG2a) isotype control, 2 µg mouse monoclonal anti-HSP90α/β clone F-8, or 10 µL hybridoma supernatant mouse monoclonal anti-WNV NS5 clone 5D4) in 500 µL total volume overnight at 4 °C with rotation. The following day, 15 µL of Protein G Dynabeads (washed 1x with 500 µL RIPA buffer prior to use) were added to the lysates (0.45 mg total beads added) and incubated for 3 h at 4 °C with rotation. Samples were subsequently applied to a magnetic rack to precipitate beads and were washed 4x with 400 µL RIPA buffer for 10 min each at RT with rotation. Protein complexes were eluted from beads and DSP crosslinker reduced via incubation for 5 min at ≈95 °C in 35 µL loading buffer (three parts RIPA buffer to one part 4X Laemmli Sample Buffer + 10% β-mercaptoethanol (Bio-Rad)). Samples were then analyzed by Western blot as appropriate, with 10 µL immunoprecipitate loaded per lane.

### 2.12. Inhibition of HSP90 in Replicon Cells and During WNV and ZIKV Infection

Seeded Huh7 WNrep cells were treated with DMSO, or with 1 μM of either geldanamycin (GA; Cayman Chemical, reconstituted in DMSO), EC144 (Tocris, Bristol, UK; reconstituted in DMSO), or NITD008 (Tocris, reconstituted in DMSO) for 12 and 24 h prior to lysis for RNA and protein analysis, or for 24 h prior to fixation and staining for immunofluorescence assay.

For HSP90 inhibition during infectious WNV and ZIKV replication, seeded Vero cells were first infected with viruses at MOI = 5 for 12 h to establish logarithmic replication. Mock- or virus-infected cells were then treated with DMSO or 1 μM HSP90 inhibitors for a further 12 h prior to lysis and analysis via Western blot.

### 2.13. Proteasome Inhibition with MG-132

A549 cells were mock-infected or infected with WNV-TX at MOI = 5. At 18 h post-infection (hpi), cells were treated with either DMSO or 40 μM MG-132 (Sigma) for a further 6 h prior to lysis and analysis via Western blot.

### 2.14. Generation of CRISPR-Targeted IFNAR1^-/-^ PH5CH8 Cells

For clustered regularly interspaced short palindromic repeats (CRISPR)/Cas9 targeting of IFNAR1, we generated the plasmid pRRL-MND-IFNAR1-2A-Puro by in-fusion cloning of the IFNAR1 genomic RNA (gRNA) sequence: 5’-GACCCTAGTGCTCGTCGCCGTGG-3’ into pRRL-MND-2A-Puro. PH5CH8 cells were transfected using the Amaxa 96-well Nucleofector Kit SF according to manufacturer’s instructions. Then, 48h post-transfection, cells were selected in growth medium with 2 μg/mL of puromycin. Gene targeting was confirmed by T7 endonuclease I assay, loss of IFNAR1 cell surface expression by flow cytometry, and loss in the response to type I IFN treatment.

### 2.15. Generation of Huh7 WNrep Cells and Replicon-Cured Cells

BHK-21 cells harboring the WNV subgenomic replicon Rluc/NeoRep were kindly provided by Pei-Yong Shi (Lo et al., 2003). Rluc/NeoRep contains a dual reporter system and was derived from the parental WNV Replicon by replacing most of the structural gene region (nt 190 to 2379) with an in-frame Renilla luciferase (Rluc) reporter gene. A neomycin phospho-transferase (Neo) gene under the control of an EMCV IRES was placed just downstream from the NS5 gene in the 3’-untranslated region (UTR). Total RNA was isolated from these cells with TRIzol according to the manufacturer’s instructions and stored at −80 °C. Rluc/NeoRep RNA was treated with DNaseI (Ambion, Austin, TX, USA). Huh7 cells were transfected with 1, 2, or 4 µg of DNaseI-treated Rluc/NeoRep RNA using Transmessenger Transfection Reagent (QIAGEN) according to the manufacturer’s instructions. After 3 h, transfection mix was replaced with cDMEM for 16-20 h at 37 °C. Cells were then washed in 1X PBS, trypsinized, transferred to new plates in complete Dulbecco’s Modified Eagle Medium (cDMEM), and allowed to recover for 48 h at 37 °C. After recovery, cDMEM was replaced with cDMEM containing G418 (G418 DMEM, 400 µg/mL) for selection of resistant colonies. Resistant colonies were transferred to new plates via colony selection discs and expanded in the presence of G418 (400 µg/mL). Approximately 11 weeks after RNA transfection, WNV Rluc/NeoRep protein expression was examined by Western blot analysis. Renilla luciferase activity of cells was also confirmed. Several distinct clones of WNV Rluc/NeoRep with variable protein and luciferase expression were recovered in Huh7 cells, with clone #8 used in these experiments.

To create a Huh7-derived control cell line for WNV Rluc/NeoRep clone #8, this line was subjected to curing with IFN. Briefly, the replicon cell line was passaged in the presence of pegylated IFNα-2b (PEG-INTRON, Schering, Berlin, Germany) at a concentration of 100 U/mL in cDMEM. After 11–25 days of maintenance in PEG-INTRON, cells were collected and analyzed for Renilla luciferase activity. When Renilla luciferase activity was below the background level of Huh7 control cells, the cured replicon cells were switched to cDMEM. Loss of the WNV replicon was confirmed by re-exposing the cells to lethal G418 to confirm death, and by RT-PCR analysis of RNA.

### 2.16. Quantification and Statistical Analysis

Statistical analyses were performed as indicated in figure legends, using GraphPad Prism version 8 software.

## 3. Results

### 3.1. WNV and ZIKV Broadly Inhibited JAK/STAT Signaling Following Cytokine Stimulation of Target Cells

**Figure 1:**
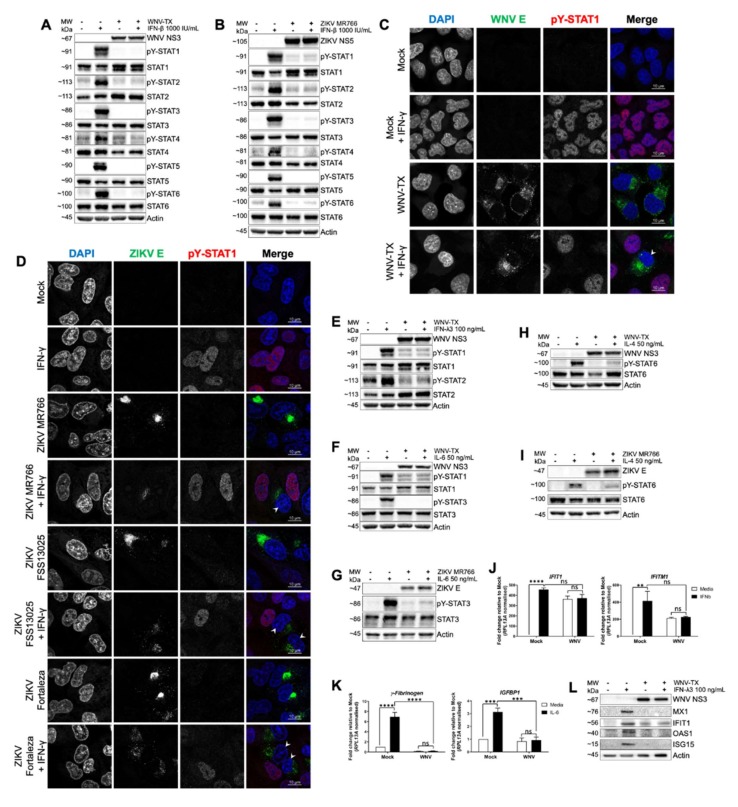
West Nile virus (WNV) and *Zika virus* (ZIKV) infections inhibit pY-STAT and gene expression in response to multiple cytokines. pY-STAT responses were inhibited in WNV- and ZIKV-infected A549 cells at 30 min post-treatment with (**A**,**B**) inferno (IFN)β, (**C**,**D**) 10 ng/mL IFNγ (arrow heads), (**E**) IFNλ3, (**F**,**G**) interleukin (IL)6, and (**H**,**I**) IL4. WNV-infected A549 cells did not respond to 17 h treatment with (**J**) IFNβ (*IFITM1* and *IFIT1* induction) or (**K**) IL6 (*γFibrinogen* and *IGFBP1* induction). (**L**) WNV-infected PH5CH8 IFNAR1^−/−^ cells did not respond to 24 h IFNλ3 treatment via expression of the interferon stimulated genes MX1, IFIT1, OAS1, and ISG15. All infections were MOI = 5 for 24 h prior to cytokine treatment unless otherwise stated. All data represent three independent experiments or are a combination of three experiments (mean ± standard error of the mean (SEM)). See also Figure S1. **** = *p* < 0.0001, *** = *p* < 0.001, ns = *p* > 0.05. Two-way ANOVA with Tukey’s posttests.

To determine the breadth of flavivirus-mediated inhibition of JAK/STAT signaling, we first assessed acute responses to JAK/STAT-dependent cytokine treatment in cells infected with WNV or ZIKV. We conducted immunoblot analyses of cytokine-induced pY-STAT responses to IFNβ for all six STAT family members in WNV- and ZIKV-infected A549 (human lung epithelial) cells (Figure 1A,B). Mock infected cells responded to IFNβ with pY of all STATs by 30 min post-treatment. In contrast, WNV- and ZIKV-infected cells were unable to mediate pY-STATs in response to high dose IFNβ (Figure 1A,B). Immunofluorescence revealed nuclear pY-STAT1 did not accumulate in response to IFNγ in WNV- and ZIKV-infected A549 cells, though pY-STAT1 did accumulate in adjacent non-infected bystander cells (Figure 1C,D). The attenuated response to IFNγ-induced pY-STAT1 was confirmed by immunoblot of WNV-infected cells (Figure S1A). WNV-infected cells also showed inhibited pY-STAT1 and 2 in response to IFNλ3 (Figure 1E). Moreover, WNV and ZIKV infection each led to inhibition of pY-STAT1 and 3 in response to human and mouse IL6 (Figure 1F,G and Figure S1B,C), and of pY-STAT6 following IL4 treatment (Figure 1H,I). Immunofluorescence also showed that WNV-infected THP-1 macrophages failed to accumulate pY-STAT1 and 3 in response to IFNβ and IL10, respectively (Figure S1D,E). Thus, WNV and ZIKV impose a broad blockade to pY-STAT mediated by several unrelated cytokines, implying one or more common features of JAK/STAT signaling could be dysregulated during flavivirus infection. Of note, background levels of pY-STAT1 and total levels of STAT1 and STAT2 were observed as being increased following virus infection in several analyses (Figure 1A,B,E,F and Figure S1A). These observations were fully expected in IFN-competent A549 cells, as IFN signaling is only blocked by flaviviruses after approximately 20–24 hpi [17]. Early antiviral signaling would then be expected induce some pY-STAT1 and ISGs (including total STAT1 and STAT2); importantly, however, neither of these were further increased upon exogenous cytokine stimulation, indicative of the virus-imposed JAK/STAT inhibition by 24hpi.

This blockade to pY-STAT corresponded to inhibition of cytokine-responsive gene induction. Analysis of endogenous interferon-induced protein with tetratricopeptide repeats 1 (*IFIT1*) and interferon-induced transmembrane protein 1 (*IFITM1*) expression in cells responding to IFNβ (Figure 1J) showed that WNV blocked induction of these ISGs in response to the cytokine (virus-induced ISG mRNA remaining from early antiviral signaling prior to establishment of JAK/STAT inhibition was not further increased). Additionally, WNV blocked induction of *γFibrinogen* and insulin-like growth factor-binding protein 1 (*IGFBP1*) in response to IL6 (Figure 1K). To assess the response of WNV-infected cells to IFNλ3, we treated human epithelial cells (hepatocytes) specifically lacking functional IFNα/β receptor expression (PH5CH8 IFNAR1^-/-^ cells) and measured expression of ISG proteins MX dynamin like GTPase 1 (MX1), IFIT1, 2´5´-oligoadenylate synthetase 1 (OAS1), and ISG15. WNV infection abrogated induction of each ISG by IFNλ3 (Figure 1L).

Importantly, NF-κB signaling in response to IL1β treatment in WNV-infected A549 cells was intact, with comparable cytokine-responsive inhibitor of κB alpha (IκBα) degradation and pS-p65 induction (Figure S1F). Upstream signaling components in this non-JAK/STAT pathway were also largely unaffected by infection (Figure S1G).

### 3.2. Cell-Intrinsic WNV NS Proteins mediated Broad JAK/STAT Inhibition

**Figure 2:**
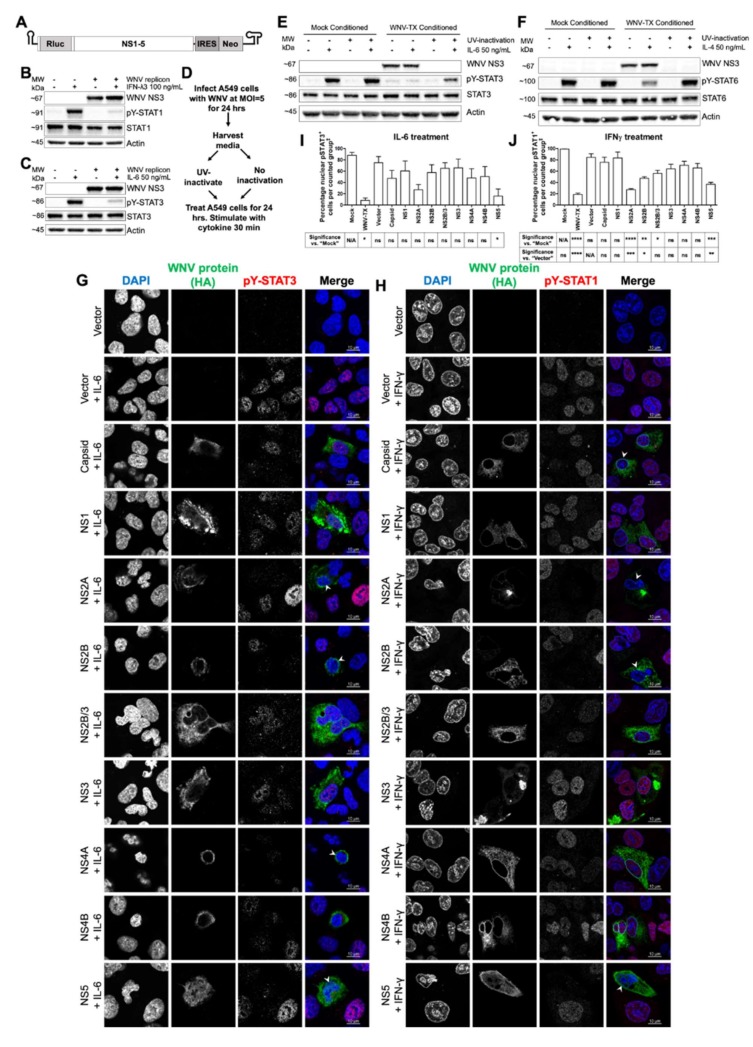
WNV nonstructural proteins were found to be responsible for Janus kinase (JAK)/STAT signaling inhibition intrinsically within infected cells. (**A**) Huh7 WNV replicon cells showed low pY-STAT responses to (**B**) IFNλ3 and (**C**) IL6 compared to cured controls. (**D**) Methodology to generate infectious and non-infectious WNV-conditioned media. Blockade of pY-STAT in response to (**E**) IL6 and (**F**) IL4 was only observed with infectious conditioned media. Recombinant expression of WNV C and nonstructural (NS) proteins in A549 cells showed differential inhibition of pY-STAT responses to (**G**,**I**) 200 ng/mL IL6 and (**H**,**J**) 10 ng/mL IFNγ (arrow heads), with WNV NS5 significantly responsible for JAK/STAT inhibition across both cytokine treatments. All cytokine treatments were for 30 min. All data represent three independent experiments or are a combination of three experiments (mean ± SEM) except “Vector” in (**I**), and “Mock” and “Vector” in (**J**) (*n* = 2). See also Figure S2. **** = *p* < 0.0001, *** = *p* < 0.001, ** = *p* < 0.01, * = *p* < 0.05, ns = *p* > 0.05. One-way ANOVA with Tukey’s posttests. ‡ “Counted group” = total cell population in several fields per experiment as measured by 4’,6-diamidino-2-phenylindole dihydrochloride (DAPI) for “Mock” and “Vector”, all WNV E-positive cells for “WNV”, and all hemagglutinin epitope tag (HA)-positive cells for WNV proteins. The red pY-STAT3 channel for images in (**G**) was enhanced equally post-acquisition across all samples to aid visual clarity.

To define which factors produced during WNV infection contributed to JAK/STAT signaling inhibition, we examined the virus replication cycle using two complementary approaches (Figure 2A–F). Huh7 hepatoma cells stably harboring a WNV replicon [31] (Figure 2A) were used to assess the JAK/STAT pathway in the context of NS protein expression and viral RNA replication, but in the absence of structural proteins. Treatment with IFNλ3 (Figure 2B) showed pY-STAT1 was reduced in WNV replicon cells compared with cells cured of replicon. Similarly, pY-STAT3 abundance was diminished in IL6-treated WNV replicon cells compared to cured controls (Figure 2C).

To assess virion impact on JAK/STAT signaling, we exposed cells to UV-inactivated WNV present in conditioned culture supernatant (Figure 2D). This approach allowed us to assess the JAK/STAT inhibitory role of viral factors such as virions, subviral particles, and NS1 protein released from infected cells [32], as well as host cytokines and metabolites produced in response to infection. Cells were first exposed to UV-inactivated WNV-conditioned media and then treated with either IL6 (Figure 2E) or IL4 (Figure 2F). Immunoblot analysis showed that cytokine treatment induced pY-STAT3 and 6 to levels comparable with control cells treated with conditioned media from mock infections. By comparison, cells exposed to supernatants containing live WNV exhibited a block of cytokine-induced pY-STAT (Figure 2E,F). Thus, extracellular factors secreted from WNV-infected cells are dispensable for the JAK/STAT signaling blockade, whereas one or more viral NS proteins are likely responsible for the broad block to pY-STAT.

To identify viral NS protein(s) responsible for JAK/STAT signaling blockade, we independently expressed each NS protein in cells and assessed their capacity to inhibit pY-STAT3 in response to IL6 (Figure 2G,I) and pY-STAT1 in response to IFNγ (Figure 2H,J) by immunofluorescence assay. As controls, we also assessed pY-STAT in cells expressing WNV capsid protein or vector alone. Expression of viral proteins themselves did not induce pY-STAT in resting cells without cytokine treatment (Figure S2). We found that among WNV NS proteins, expression of NS5 caused significant inhibition of pY-STAT accumulation in cells treated with either cytokine. Expression of NS2A, NS2B, and NS2B/3 showed significant inhibition of pY-STAT1 following treatment with IFNγ only. Expression of any individual NS protein was unable to block JAK/STAT signaling as efficiently as live WNV, suggesting viral NS proteins likely act in a complementary manner to confer broad JAK/STAT antagonism.

### 3.3. Flavivirus Infection Lead to JAK and Cytokine Receptor Degradation by the Proteasome

**Figure 3:**
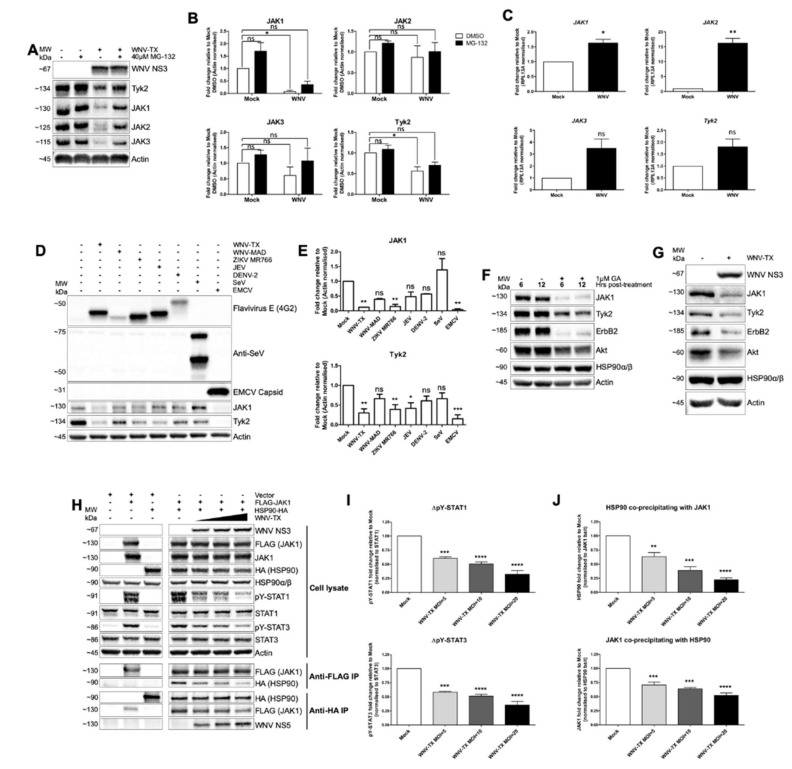
Flavivirus infection led to loss of abundance of heat shock protein 90 (HSP90) client kinases including JAK proteins, with interaction between HSP90 and JAK1 disrupted. (**A**,**B**) JAK family proteins are decreased in abundance during WNV infection. Treatment with MG-132 at 18 h post-infection (hpi) partially recovered JAK abundance in WNV-infected cells. (**C**) Transcripts of JAK1, JAK2, JAK3, and Tyk2 increased during WNV infection. (**D**,**E**) Infection of A549 cells with WNV-TX, ZIKV MR766, Japanese encephalitis virus (JEV), and encephalomyocarditis virus (EMCV) led to loss of Tyk2 and (for all except JEV) JAK1 by 36 hpi. Infection with less robustly infecting flaviviruses WNV-MAD and dengue virus (DENV)-2, as well as with Sendai virus (SeV), did not lead to significant protein loss. (**F**) Treatment of A549 cells with HSP90 inhibitor geldanamycin (GA) for 6 and 12 h diminished abundance of JAK1, Tyk2, erythroblastosis oncogene B2 (ErbB2), and AKR mouse strain thymoma-related protein (Akt). (**G**) HSP90 client kinases ErbB2 and Akt displayed decreased abundance in WNV-infected A549 cells in a similar manner to JAK1 and Tyk2 by 24 hpi. (**H**–**J**) HEK-293T cells mock- or WNV-infected (MOI = 5, 10, and 20) were co-transfected with FLAG-epitope tagged JAK1 (FLAG-JAK1) and HSP90-HA. (**H**,**I**) Transfection of JAK1 was sufficient for pY-STAT1 and three responses, and this pY-STAT was significantly blocked with increasing WNV. (**J**) Reciprocal HA- and FLAG-tagged co-immunoprecipitation (co-IP) showed significantly decreased interaction of co-precipitated HSP90 and JAK1 with increasing WNV. All infections were in A549 cells at MOI = 5 for 24 h prior to cytokine treatment unless stated, except SeV which was infected at 40 hemagglutination units per well. All data represent three independent experiments or are a combination of three experiments (mean ± SEM), except (**G**) (*n* = 2). **** = *p* < 0.0001, *** = *p* < 0.001, ** = *p* < 0.01, * = *p* < 0.05, ns = *p* > 0.05. Paired *t*-tests (**C**). One-way ANOVA with Dunnett’s Multiple Comparison post-test (**I**, **J**). Irrelevant lanes were cropped between controls and infected samples in (**H**).

Considering the fact that the broad inhibition of pY-STAT responses and gene induction by WNV and ZIKV appeared to be unrelated to the identity of the cytokine used to stimulate cells (Figure 1), we sought to determine whether infection with these viruses affected JAK proteins proximal to these events in the JAK/STAT signaling cascade. Immunoblot of WNV-infected A549 cells treated under control conditions (DMSO) demonstrated that at 24 hpi, all JAK family members (JAK1-3 and Tyk2) had reduced protein abundance compared to mock-infected cells. This loss of abundance was partially rescued by treatment with the proteasome inhibitor MG-132 for 6 h, starting at 18 hpi. MG132 led to increased JAK family protein abundance in WNV-infected cells to levels similar to mock, DMSO-treated cells (Figure 3A,B). Time course analyses demonstrated that loss of JAK1 abundance could be observed from approximately 16 hpi (Figure S1H). To assess whether JAK loss was in part due to RNA regulation, we quantified *JAK1*, *JAK2*, *JAK3*, and *Tyk2* transcripts within mock- and WNV-infected cells at 24 hpi (Figure 3C). WNV infection led to induction of JAK mRNA, and thus loss of JAK abundance is likely mediated solely by post-transcriptional processes.

To determine if loss of JAK family protein abundance was a phenotype extending beyond the virulent WNV strain we predominantly used, we evaluated the abundance of JAK1 and Tyk2 in A549 cells infected with a panel of flaviviruses, as well as control viruses including Sendai virus (SeV) and encephalomyocarditis virus (EMCV), at 36 hpi (Figure 3D,E). Compared to mock-infected cells, infection with WNV-TX, WNV-MAD, ZIKV MR766, JEV, and DENV-2 all showed a trend of variably decreased JAK1 and Tyk2 abundance (Figure 3D). Quantification of band intensity across three independent experiments demonstrated that significant decreases in both JAK1 and Tyk2 occurred during WNV-TX and ZIKV MR766 infection (Figure 3E), whereas only decrease in Tyk2 abundance was significant for JEV. Despite the trend towards decreased abundance of these JAK family proteins, quantification from WNV-MAD and DENV-2 infection (both viruses that replicate less robustly in A549 cells) demonstrated that these decreases were not significant. EMCV infection (which shuts down host translation) [33] led to significant loss of JAK1 and Tyk2. In contrast, SeV infection displayed only slight alteration in abundance of these proteins. Therefore, loss of JAK protein abundance is a feature common to several flavivirus infections.

### 3.4. NS5 Disrupted HSP90-Client Kinase Interaction to Block JAK/STAT Signaling

To identify the mechanism WNV NS5 uses to drive JAK/STAT inhibition, we evaluated cellular processes known to regulate JAK stability and cytokine-responsive pY-STAT. As HSP90 is required to mediate the correct kinase-active conformational folding and stability of JAK proteins (and other specific client kinases) [34,35], we reasoned that viral dysregulation of HSP90 might confer reduced abundance of JAKs and other HSP90 clients within infected cells. Indeed, we found that chemical inhibition of HSP90 activity with geldanamycin (GA) led to loss in abundance of JAK1 and Tyk2, as well as other HSP90 client kinases Akt and ErbB2 at 6 and 12 h post-treatment (Figure 3F). Importantly, we were able to see an analogous loss in abundance in all four of these HSP90 client kinases at 24 hpi with WNV (Figure 3G), directly phenocopying chemical HSP90 inhibition. Thus, viral dysregulation of HSP90 might be responsible for the broad block to pY-STAT during WNV infection.

We directly assessed the effect of WNV infection on the interaction of HSP90 with JAK1 using overexpression of epitope-tagged proteins and co-immunoprecipitation (co-IP) (Figure 3H,J) from virus infected cells. HEK-293T cells were mock- or WNV-infected at MOI = 5, 10, and 20. At 2 hpi, cells were co-transfected with plasmids encoding FLAG-JAK1 and HA-HSP90α (with vector controls). Immunoblot of whole cell lysate (Figure 3H) showed that transfection of FLAG-JAK1 was sufficient to drive ligand- and receptor-independent pY-STAT1 and 3 by 24 hpi. Increased WNV MOI led to significantly decreased levels of pY-STAT (Figure 3I), without loss in abundance of ectopic, overexpressed JAK1. These results demonstrated a fundamental point—degradation of JAK1 is not required per se for pY-STAT inhibition. Degradation presumably occurs subsequent to the mechanism rendering kinase activity of JAKs defective.

Reciprocal co-IP of JAK1 and HSP90 (Figure 3J) revealed the mechanism leading to kinase-inactive JAK proteins. Increasing infection with WNV led to significantly decreased interaction between JAK1 and HSP90. Presumably, this diminished interaction prevented HSP90 from properly chaperoning JAK1 to promote an active conformational fold. Importantly, we found this decline in JAK1-HSP90 interaction occurred concomitant with increasing interaction between HSP90 and viral NS5 (Figure 3J). Thus, the precise mechanism of pY-STAT inhibition involves WNV NS5 targeting of HSP90 to disrupt its interaction with JAKs, preventing the JAK activity that drives pY-STAT (Figure 3H,I), and leading eventually to JAK proteasomal degradation (see Figure 3A,B,D,G).

### 3.5. HSP90 Antagonism Was Found to be Linked to Interaction with Viral NS5 at Sites of RNA Replication

**Figure 4:**
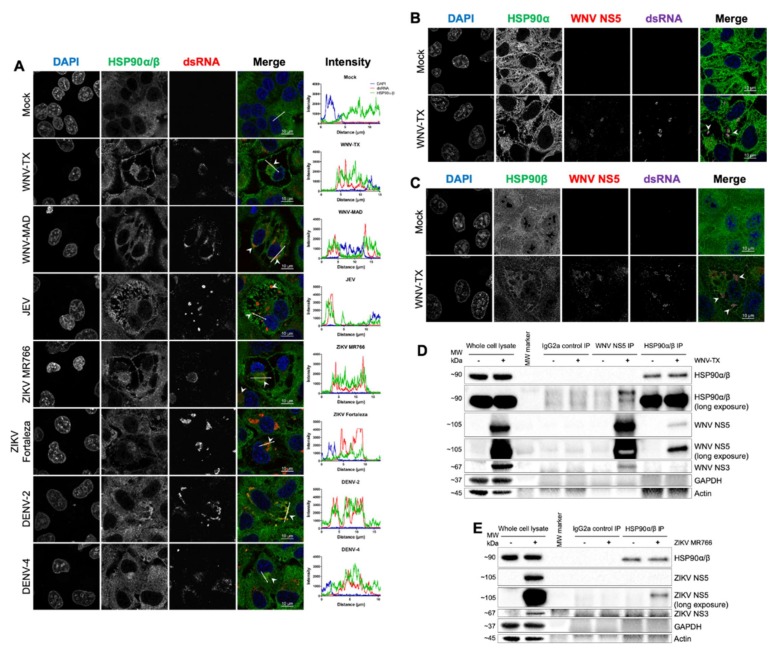
Flavivirus NS5 interacted with HSP90 at sites of viral RNA replication. (**A**) HSP90 co-localized with double-stranded RNA (dsRNA) at 24 hpi with WNV-TX, WNV-MAD, JEV, ZIKV MR766, ZIKV Fortaleza, DENV-2, and DENV-4 (arrow heads). WNV NS5 co-localized with both (**B**) HSP90α and (**C**) HSP90β and dsRNA in infected A549 cells (arrow heads). (**D**) ReCLIP experiments with WNV-infected A549 cells showed NS5 co-precipitation with HSP90, and HSP90 was recovered following NS5 IP. Controls showed NS3 was detected following NS5 IP (replication complexes), and neither glyceraldehyde 3-phosphate dehydrogenase (GAPDH) nor Actin were co-precipitated under any condition. The IgG2a isotype control failed to IP any analyzed protein. (**E**) ReCLIP analysis of ZIKV-infected cells showed an analogous NS5–HSP90 interaction. All infections were at MOI = 5 for 24 h (except DENV-4 where MOI = 3). All data represent three independent experiments, except (E) (*n* = 2).

To examine the context of virus-induced changes to HSP90 function, we examined localization of this chaperone within flavivirus-infected A549 cells at 24 hpi. Immunofluorescence showed HSP90 co-localized with or is adjacent to double-stranded RNA (dsRNA) within replication complexes of WNV-TX, WNV-MAD, JEV, ZIKV MR766, ZIKV Fortaleza, DENV-2, and DENV-4 (Figure 4A). High-resolution imaging showed both isoforms HSP90α and HSP90β co-localized with WNV NS5 and dsRNA (Figure 4B,C and Figure S3A,B).

We conducted analyses to interrogate the interaction of endogenous HSP90 and NS5 in WNV- and ZIKV-infected cells using reversible cross-linked IP (ReCLIP). Endogenous HSP90 bound to virus-produced WNV NS5 in reciprocal co-IP analyses (Figure 4D). Similarly, ZIKV NS5 was found in complex with endogenous HSP90 in infected cells (Figure 4E). The HSP90–NS5 interaction was specifically recovered by anti-NS5 or anti-HSP90, as the NS5-HSP90 complex was not recovered using isotype control IgG2a antibody. We confirmed specific interaction of ZIKV NS5 with HSP90 via further analysis of FLAG-tagged ZIKV NS5 in HEK-293T cells and co-IP of HSP90 (Figure S3C). ZIKV NS5 could co-IP endogenous HSP90, however, neither vector nor ZIKV capsid protein conferred HSP90 interaction.

### 3.6. HSP90 Activity Was not Required to Support Flavivirus RNA Replication nor to Stabilize Viral Proteins

**Figure 5:**
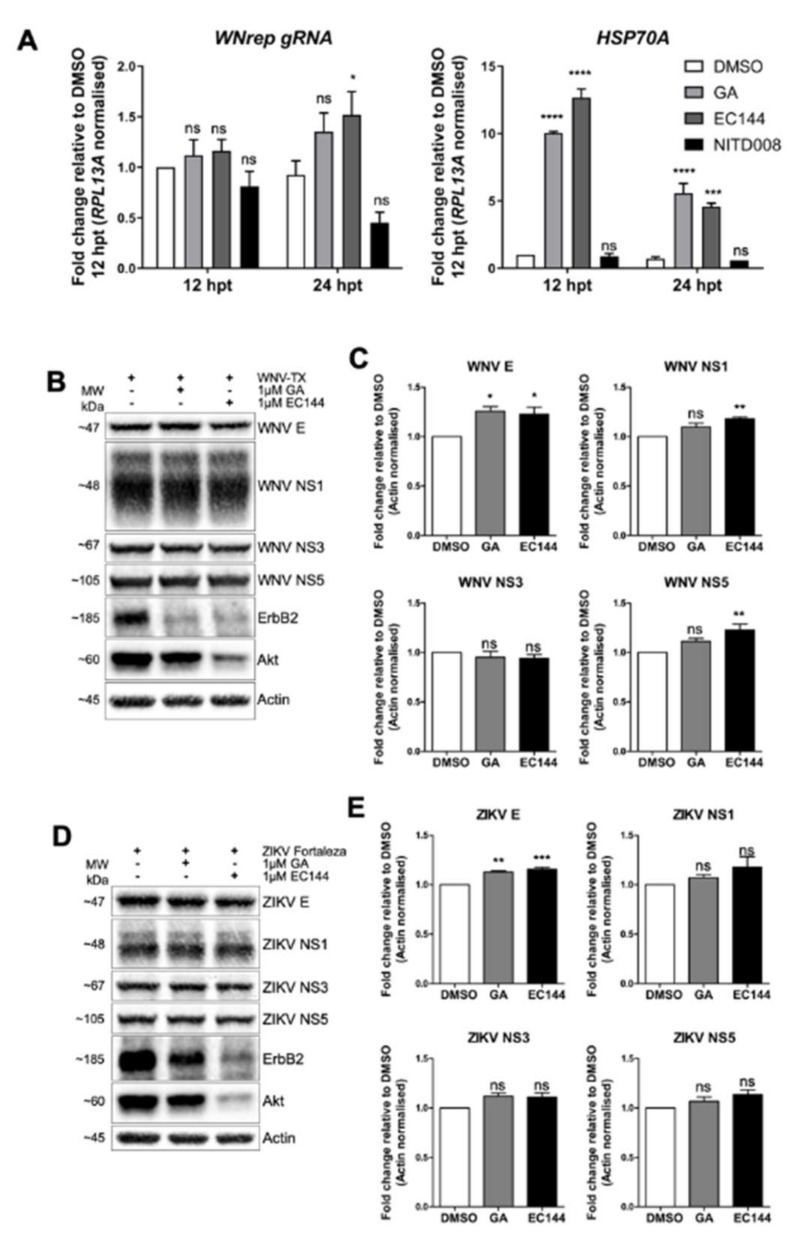
Flavivirus RNA replication and protein stability did not require HSP90 activity. (**A**) Huh7 WNV replicon cells were treated with HSP90 inhibitors GA and EC144, or nucleoside analogue NITD008 for 12 and 24 h to assess effects on RNA replication. Analysis of WNV replicon RNA (WNrep genomic RNA (gRNA)) revealed increased abundance following HSP90 inhibitor treatment, in contrast to reduced gRNA following NITD008. This was coincident with significant induction of HSP70A mRNA following HSP90 inhibitor treatment. Vero cells 12 hpi with (**B**,**C**) WNV or (**D**,**E**) ZIKV at MOI = 5 were treated with DMSO or HSP90 inhibitors for a further 12 h. (**B**,**D**) HSP90 inhibitors reduced Akt and ErbB2 abundance, and changes to viral proteins were not detected. Quantities of (**C**) WNV E, NS1, and NS5, as well as (**E**) ZIKV E were moderately yet significantly increased upon HSP90 inhibition. All data represent three independent experiments or are a combination of three experiments (mean ± SEM), with the exception of HSP70A mRNA quantification in (**C**) (*n* = 2). **** = *p* < 0.0001, *** = *p* < 0.001, ** = *p* < 0.01, * = *p* < 0.05, ns = *p* > 0.05. Two-way repeated measures ANOVA with Holm–Sidak’s post-tests (**A**), and one-way ANOVA with Dunnett’s multiple comparison post-tests (**C**,**E**).

We considered the possibility that flaviviruses may usurp HSP90 to promote viral RNA replication and viral protein function, as has been reported for flavivirus interactions with HSP70 [26,27,28]. To address this notion, we assessed the effect of chemical HSP90 inhibition upon the abundance of viral RNA and proteins, and on dsRNA NS protein localization within Huh7 WNV replicon cells. Compared to DMSO-treated control cells, WNV replicon cells 24 hpt with HSP90 inhibitors GA and EC144 showed stable co-localization of NS3 and NS5 by immunofluorescence analysis (Figure S4A). However, upon HSP90 inhibition in treated cells, NS1 and dsRNA shifted from co-localization with the NS proteins to distribute as bright foci directly adjacent to NS3 and NS5 (Figure S4A,B). Changes in abundance of replicon antigens was undetectable under conditions of HSP90 inhibition in treated cells. In contrast, a decrease in intensity was observed for controls treated with the flavivirus replication inhibitor NITD008. Analysis of replicon genomic RNA (WNrep gRNA; Figure 5A) showed HSP90 inhibition via EC144 treatment significantly increased gRNA abundance by 24 h. In contrast, NITD008 treatment led to decreased gRNA. Analysis of HSP70A mRNA showed significant induction of this chaperone upon HSP90 inhibition as expected [36], but not following NITD008 treatment.

We also measured the abundance of WNV replicon NS proteins at 12 and 24 hpt by immunoblot analysis (Figure S4C,D). Though reduced abundance of Akt and ErbB2 were found upon GA and EC144 treatment, neither inhibitor caused a decrease in replicon NS proteins. This contrasts with significant 30–50% reduction of NS proteins with NITD008. We also examined the impact of HSP90 inhibition upon protein abundance during live WNV and ZIKV infection. Vero cells 12 hpi were treated with DMSO, GA, or EC144 for a further 12 h. For both viruses, HSP90 inhibitors decreased Akt and ErbB2 abundance, though little change to viral proteins was detected (Figure 5B,D). Quantification of band intensity showed that WNV and ZIKV protein levels did not decrease during HSP90 inhibition (Figure 5C,E). Indeed, WNV E, NS1, NS5, and ZIKV E were significantly increased upon HSP90 inhibitor treatment, as similarly shown for DENV infection [37]. Taken together, these observations indicate that flavivirus replication is not dependent upon HSP90 activity.

## 4. Discussion

Our detailed pathway analyses revealed the fact that flavivirus-directed JAK/STAT signaling inhibition extends far beyond classical IFN responses, with antagonism of this pathway found in response to every cytokine we investigated. We proposed a mechanistic model of flavivirus JAK/STAT antagonism in which flavivirus NS5 (possibly in conjunction with other NS proteins) interacts with host HSP90 at the site of viral RNA replication. As a consequence of NS5 interaction, the HSP90-kinase client homeostasis is disrupted, leading to inappropriate chaperoning of JAK proteins, loss of their activity, protein kinase instability, and subsequent degradation via the proteasome. This process reduces abundance of JAKs, with the remaining pool of kinases lacking chaperone-supported enzymatic activity. Inactive JAKs cannot transmit pY to STAT proteins, and thus infected cells become refractory to cytokines that signal through the JAK/STAT pathway, including IFNs, proinflammatory cytokines, and immune regulatory cytokines.

HSP90 has been well described as a regulator of JAK activity and stability [34,35,38]. Inhibition of HSP90 leads to degradation of JAK1 and JAK2 to attenuate the pY-STAT1 and 2 response to IFNβ and IFNγ [34]. In Hodgkin lymphoma cells, inhibition of HSP90 led to loss of JAK1, JAK3, and Tyk2, and constitutive pY of STAT1, 3, 5, and 6 was ablated [35]. Thus, HSP90 inhibitors phenocopy JAK/STAT antagonism by flaviviruses, a similarity of function that we confirmed with side-by-side analyses. Our data revealed for the first time that flavivirus NS5 binds HSP90 to mediate broad STAT inhibition, which extends upon descriptive reports from other groups that identified JEV, DENV, and ZIKV NS5 interaction with HSP90, but did not define the relevance of this interaction [39,40,41]. Several DENV proteins have also been shown to bind HSP90 [37]. Our demonstration of JAK destabilization as a consequence of HSP90–NS5 interaction provides important functional insight into viral evasion of host innate immunity and inflammatory signaling. Moreover, we showed that Akt and ErbB2 levels decrease during flavivirus infection as a result of NS5-HSP90 interaction, implying that processes mediated by these and other HSP90-client kinases are likely altered during infection, possibly contributing to viral pathogenesis.

We showed that HSP90 chaperone activity is not required for flavivirus replication or protein stability but that HSP90 inhibition leads to slight enhancement of infection. This contrasts with a recent report showing activity of grp94 (an endoplasmic reticulum-resident paralog of HSP90) is required for DENV and ZIKV infection [42]. Vero cells were specifically chosen for these experiments as they lack IFN production [43,44], and thus the moderately enhanced viral growth that we observed was unlikely due to further dysregulation of antiviral signaling. Rather, we propose that induction of HSP70 likely contributes to the greater WNV and ZIKV replication seen here upon HSP90 inhibition, as HSP70 is known to enhance flavivirus replication [26,27,28]. Thus, flavivirus interaction with HSP90 may represent a common strategy among flaviviruses to dysregulate and suppress host IFN antiviral signaling rather than directly supporting viral replication and protein function. The broad inhibition of pY-STAT resulting from the NS5-HSP90 interaction then imparts collateral dysregulation of IFN-independent cytokine actions.

The remarkable breadth of cytokine signaling inhibition imposed by the NS5–HSP90 interaction may have consequences for flavivirus disease phenotypes outside of the context of innate antiviral defenses. In the context of an acute immune response, flavivirus-infected myeloid cells (important targets of infection) [13,45,46] would be less responsive to anti-inflammatory IL10 and IL4 signaling through STAT3 and 6, respectively, perhaps altering the immune phenotype polarization of myeloid cells and enhancing immune-mediated tissue damage. Additionally, reduced response of flavivirus-infected antigen-presenting cells to IFNγ through pY-STAT1 may affect adaptive immunity priming actions to alter T cell-mediated immunity.

Our findings of HSP90 interaction share similarities with recent reports on involvement of HSP70 in flavivirus infection and host inflammatory signaling [26,27,28], suggesting chaperones are key regulators of pathogenesis. Flaviviruses productively infect and transmit between diverse hosts from arthropods (ticks and mosquitoes) to vertebrates (birds and mammals) [47]. Hence, flaviviruses must exploit diverse systems to promote replication; a setting that may favor the targeting of factors conserved between reservoirs and vectors. HSP70 and HSP90 are highly conserved between these organisms, both in structure and function [48,49], and thus it is feasible that strategies developed to exploit these chaperones could be viable across diverse hosts. Indeed HSP70 inhibitors antagonize flavivirus replication in both insect and mammalian cells [26,27], and JAK/STAT inhibition by flaviviruses in mosquito cells is dependent on NS5 and involves proteasomal activity [50]. Thus, we propose flaviviruses target HSPs to facilitate infection in diverse hosts, with HSP70 and grp94 [42] activity usurped for viral replication and HSP90 activity disrupted to antagonize innate immune defense. This division of functions regarding flavivirus interactions with cytoplasmic HSPs (i.e., interaction with HSP70 to promote viral protein folding vs. targeting of HSP90 to disrupt host immunity) is striking. These chaperones normally operate in close cooperation with one another [49], and thus this distinct separation of roles warrants further investigation to discern the consequences of this division for host cell function. Whether a HSP antagonism strategy is utilized to promote pathogenesis of other virus groups also remains to be investigated. As chaperones support so many cellular functions, the unforeseen medical consequences of viral HSP antagonism are likely substantial.

## Supplementary Materials

The following are available online at https://www.mdpi.com/2073-4409/9/4/899/s1: Figure S1: WNV infection inhibited STAT phosphorylation in response to multiple cytokines. Figure S2: Recombinant WNV protein expression did not induce pY-STAT3 or pY-STAT1. Figure S3: HSP90 interacted with NS5 and was localized to sites of viral dsRNA. Figure S4: HSP90 inhibition did not perturb WNV replication. Table S1: Reagents and resources used.
