# Supplementary material for: Flavivirus Nonstructural Protein NS5 Dysregulates HSP90 to Broadly Inhibit JAK/STAT Signaling
Source: Cells. 2020 Apr 7;9(4):899. doi: 10.3390/cells9040899 (PMC7226784; doi:10.3390/cells9040899)
Supplement: Supplementary file 1 [file cells-09-00899-s001.zip › Roby et al - Cells - Supplementary table S1 26-2-20.docx]

**Table S1.** Reagents and resources used.

| **Reagent/Resource** | **Source** | **Identifier** |
| --- | --- | --- |
| Antibodies |  |  |
| Mouse monoclonal anti-Actin clone C4 | Millipore | Cat#MAB1501; RRID: AB_2223041 |
| Rabbit polyclonal anti-Akt | Cell Signaling Technology | Cat#9272; RRID: AB_329827 |
| Mouse monoclonal anti-dsRNA clone J2 | SCICONS | Cat#10010200; RRID: AB_2651015 |
| Rabbit polyclonal anti-EMCV Capsid | Ann C. Palmenberg | N/A |
| Rabbit monoclonal anti-ErbB2 | Cell Signaling Technology | Cat#4290; RRID: AB_10828932 |
| Mouse monoclonal anti-FLAG clone M2 | Sigma | Cat#F1804; RRID: AB_262044 |
| Mouse monoclonal anti-Flavivirus Envelope clone 4G2 | Purified from hybridoma D1-4G2-4-15 supernatant | See Cell Lines below |
| Mouse monoclonal anti-HA | Sigma | Cat#H9658; RRID:AB_260092 |
| Mouse monoclonal anti-HA-Alexa488 | Cell Signaling Technology | Cat#2350; RRID: AB_10693161 |
| Rabbit monoclonal anti-HA-Magnetic bead conjugate | Cell Signaling Technology | Cat#11846; RRID:AB_2665471 |
| Mouse monoclonal anti-HSP90α/β clone F-8 | Santa Cruz Biotechnology | Cat#sc-13119; RRID: AB_675659 |
| Rabbit monoclonal anti-HSP90α/β | Abcam | Cat#ab203126; RRID:AB_2800428 |
| Rabbit monoclonal anti-HSP90α-Alexa488 | Abcam | Cat#ab193900; RRID:AB_2800429 |
| Rabbit monoclonal anti-HSP90β-Alexa488 | Abcam | Cat#ab202822; RRID:AB_2800430 |
| Mouse monoclonal anti-IκBα | Cell Signaling Technology | Cat#4814; RRID: AB_390781 |
| Mouse monoclonal anti-IKKβ | Upstate | Cat#05-535; RRID: AB_2122161 |
| Mouse monoclonal anti-IKKγ | BD Biosciences | Cat#611306; RRID: AB_398832 |
| Rabbit polyclonal anti-IFIT1 | UT Southwestern Antibody Core facility | N/A |
| Rabbit polyclonal anti-ISG15 | Cell Signaling Technology | Cat#2743; RRID: AB_10692793 |
| Mouse monoclonal anti-JAK1 | Cell Signaling Technology | Cat#50996; RRID:AB_2716281 |
| Rabbit monoclonal anti-JAK1 | Cell Signaling Technology | Cat#3344; RRID: AB_10828108 |
| Rabbit monoclonal anti-JAK2 | Cell Signaling Technology | Cat#3230; RRID: AB_2128522 |
| Rabbit monoclonal anti-JAK3 | Cell Signaling Technology | Cat#8827; RRID: AB_11127204 |
| Rabbit polyclonal anti-MX1 | UT Southwestern Antibody Core facility | N/A |
| Rabbit polyclonal anti-MyD88 | Abcam | Cat#ab2068; RRID: AB_2146694 |
| Mouse monoclonal anti-NFκB p65 | Cell Signaling Technology | Cat#6956; RRID: AB_10828935 |
| Rabbit monoclonal anti-pS-NFκB p65 | Abcam | Cat#ab76302; RRID: AB_1524028 |
| Mouse monoclonal anti-OAS1 | Kineta | N/A |
| Rabbit polyclonal anti-Sendai Virus | Ilkka Julkunen | N/A |
| Rabbit polyclonal anti-STAT1 | Cell Signaling Technology | Cat#9172; RRID: AB_10831362 |
| Rabbit monoclonal anti-pY-STAT1 | Cell Signaling Technology | Cat#7649; RRID: AB_10950970 |
| Rabbit monoclonal anti-pY-STAT1 | Cell Signaling Technology | Cat#9167; RRID: AB_561284 |
| Rabbit monoclonal anti-STAT2 | Cell Signaling Technology | Cat#72604; RRID:AB_2799824 |
| Rabbit polyclonal anti-pY-STAT2 | Millipore | Cat#07-224; RRID:AB_2198439 |
| Rabbit monoclonal anti-STAT3 | Cell Signaling Technology | Cat#4904; RRID: AB_331269 |
| Rabbit monoclonal anti-pY-STAT3 | Cell Signaling Technology | Cat#9145; RRID: AB_2491009 |
| Rabbit polyclonal anti-pY-STAT3 | Cell Signaling Technology | Cat#9131; RRID: AB_331586 |
| Rabbit monoclonal anti-STAT4 | Cell Signaling Technology | Cat#2653; RRID: AB_2255156 |
| Rabbit polyclonal anti-pY-STAT4 | R&D Systems | Cat#AF4319; RRID:AB_884482 |
| Mouse monoclonal anti-STAT5 | Therno Fisher Scientific | Cat#33-5900; RRID:AB_2533129 |
| Rabbit monoclonal anti-pY-STAT5 | Cell Signaling Technology | Cat#4322; RRID: AB_10548756 |
| Rabbit monoclonal anti-STAT6 | Cell Signaling Technology | Cat#5397; RRID: AB_11220421 |
| Rabbit polyclonal anti-pY-STAT6 | Cell Signaling Technology | Cat#9361; RRID: AB_331595 |
| Rabbit polyclonal anti-TRAF6 | Santa Cruz Biotechnology | Cat#sc-7221; RRID: AB_793346 |
| Rabbit polyclonal anti-Tyk2 | Upstate | Cat#06-638; RRID: AB_310197 |
| Mouse monoclonal E16 anti-WNV Envelope | Michael S. Diamond | N/A |
| Humanized monoclonal E16 anti-WNV Envelope-FITC | Michael S. Diamond | N/A |
| Mouse monoclonal anti-WNV NS1 (13NS1) | Michael S. Diamond | N/A |
| Rabbit polyclonal anti-WNV NS1 | GeneTex | Cat#GTX132053; RRID:AB_2800432 |
| Mouse monoclonal anti-WNV NS3 | R&D Systems | Cat#MAB2907; RRID:AB_2215926 |
| Goat polyclonal anti-WNV NS3-biotin | R&D Systems | Cat# BAF2907; RRID:AB_2215927 |
| Mouse monoclonal anti-WNV NS5 clone 5D4 | Roy A. Hall | N/A |
| Mouse monoclonal anti-WNV NS5 clone 7G6 | Roy A. Hall | N/A |
| Mouse monoclonal anti-WNV NS5 clone 5C11 | Roy A. Hall | N/A |
| Mouse monoclonal anti-WNV NS5 clone 6A10 | Roy A. Hall | N/A |
| Rabbit polyclonal anti-WNV NS5 | GeneTex | Cat#GTX131961; RRID:AB_2800433 |
| Mouse monoclonal anti-ZIKV Envelope clone ZV-23 | Michael S. Diamond | N/A |
| Mouse monoclonal anti-ZIKV Envelope clone ZV-13 | Michael S. Diamond | N/A |
| Mouse monoclonal anti-ZIKV NS1 | Arigo | Cat#ARG65781; RRID:AB_2800434 |
| Rabbit polyclonal anti-ZIKV NS5 | GeneTex | Cat#GTX133327; RRID:AB_2800435 |
| Mouse IgG2a isotype control | BioLegend | Cat#401502; RRID:AB_2800437 |
| Donkey polyclonal anti-Mouse IgG-Alexa680 | Jackson ImmunoResearch | Cat#715-625-151 ; RRID:AB_2340869 |
| Donkey polyclonal anti-Mouse IgG-Alexa790 | Jackson ImmunoResearch | Cat#715-655-150 ; RRID:AB_2340870 |
| Donkey polyclonal anti-Mouse IgG-HRP | Jackson ImmunoResearch | Cat#715-035-150 ; RRID:AB_2340770 |
| Goat polyclonal anti-Mouse IgG-Alexa488, highly cross-adsorbed | Thermo Fisher Scientific | Cat#A-11029 ; RRID:AB_2534088 |
| Goat polyclonal anti-Mouse IgG1-Alexa488, highly cross-adsorbed | Thermo Fisher Scientific | Cat#A-21121 ; RRID:AB_2535764 |
| Goat polyclonal anti-Mouse IgG-Alexa594, highly cross-adsorbed | Thermo Fisher Scientific | Cat#A-11032 ; RRID:AB_2534091 |
| Goat polyclonal anti-Mouse IgG-HRP | Jackson ImmunoResearch | Cat#115-035-003 ; RRID:AB_10015289 |
| Goat polyclonal anti-Mouse IgG1-Alexa568, highly cross-adsorbed | Thermo Fisher Scientific | Cat#A-21124 ; RRID:AB_2535766 |
| Goat polyclonal anti-Mouse IgG2a-Alexa568, highly cross-adsorbed | Thermo Fisher Scientific | Cat#A-21134 ; RRID:AB_2535773 |
| Goat polyclonal anti-Mouse IgG2a-Alexa647, cross-adsorbed | Thermo Fisher Scientific | Cat#A-21241 ; RRID:AB_2535810 |
| Donkey polyclonal anti-Rabbit IgG-Alexa680 | Jackson ImmunoResearch | Cat#711-625-152 ; RRID:AB_2340627 |
| Donkey polyclonal anti-Rabbit IgG-Alexa790 | Jackson ImmunoResearch | Cat#711-655-152 ; RRID:AB_2340628 |
| Donkey polyclonal anti-Goat IgG-Alexa790 | Jackson ImmunoResearch | Cat#705-655-147 ; RRID:AB_2340441 |
| Donkey polyclonal anti-Goat IgG-HRP | Jackson ImmunoResearch | Cat#705-035-147 ; RRID:AB_2313587 |
| Goat polyclonal anti-Rabbit IgG-Alexa488, highly cross-adsorbed | Thermo Fisher Scientific | Cat#A-11034 ; RRID:AB_2576217 |
| Goat polyclonal anti-Rabbit IgG-Alexa568, highly cross-adsorbed | Thermo Fisher Scientific | Cat#A-11036 ; RRID:AB_10563566 |
| Goat polyclonal anti-Rabbit IgG-Alexa594, highly cross-adsorbed | Thermo Fisher Scientific | Cat#A-11037 ; RRID:AB_2534095 |
| Goat polyclonal anti-Rabbit IgG-HRP | Jackson ImmunoResearch | Cat#111-035-003 ; RRID:AB_2313567 |
| Bacterial and Virus Strains |  |  |
| DENV-2 strain New Guinea C | Alec J. Hirsch | N/A |
| DENV-4 strain H241 | Alec J. Hirsch | N/A |
| NEB® 5-alpha F'Iq Competent Escherichia coli (High Efficiency) | New England Biolabs | Cat#C2992H |
| EMCV strain Mengo infectious clone | Ann C. Palmenberg | N/A |
| JEV stain Nakayama | Michael S. Diamond | N/A |
| Sendai virus strain Cantell | Charles River | Cat#10100774 |
| WNV strain Madagascar-AnMg798 infectious clone (WNV-MAD) | Suthar et al. J Virol 2012, 86, 7704-7709. | N/A |
| WNV strain Texas-HC2002 infectious clone (WNV-TX) | Suthar et al. J Virol 2012, 86, 7704-7709. | N/A |
| ZIKV strain Brazil-Fortaleza 2015 | Michael S. Diamond | GenBank accession number: [KX811222.1](http://www-ncbi-nlm-nih-gov.offcampus.lib.washington.edu/nuccore/?term=KX811222.1) |
| ZIKV strain Cambodia-FSS13025 2010 | The World Reference Center of Emerging Viruses and Arboviruses | GenBank accession number: [KU955593](http://www-ncbi-nlm-nih-gov.offcampus.lib.washington.edu/nuccore/?term=KU955593) |
| ZIKV strain Uganda-MR766 1947 | ATCC | Cat#ATCC-VR-84 |
| Chemicals, Peptides, and Recombinant Proteins |  |  |
| Dithiobis(succinimidyl propionate) (DSP) | Therno Fisher Scientific | Cat#22586 |
| DMSO | Sigma | Cat#D2650 |
| EC 144 | Tocris | Cat#4701 |
| G418 | Corning | Cat#61-234-RG |
| Geldanamycin | Cayman Chemical | Cat#13355 |
| Human IL-1β | BioLegend | Cat#579404 |
| Human IL-4 | PeproTech | Cat#200-04 |
| Human IL-6 | BioLegend | Cat#570804 |
| Human IL-10 | eBioscience | Cat#14-8109-80 |
| Human IFN-β | Provided by Toray Industries | N/A |
| Human IFN-γ | R&D Systems | Cat#285-IF-100 |
| Human IFN-λ3 | R&D Systems | Cat#5259-IL-025 |
| MG-132 | Sigma | Cat#M7449-200UL |
| Murine IL-6 | BioLegend | Cat#575702 |
| NITD 008 | Tocris | Cat#6045 |
| PMA | Sigma | Cat#P8139 |
| Critical Commercial Assays |  |  |
| Lipofectamine 3000 | Thermo Fisher Scientific | Cat#L3000008 |
| RNeasy Mini Kit | QIAGEN | Cat#74104 |
| iScript cDNA synthesis kit | Bio-Rad | Cat#1708897BUN |
| SYBR Green PCR Master Mix | Thermo Fisher Scientific | Cat#4312704 |
| ECL prime western blotting reagent | Fisher Scientific | Cat#RPN2232 |
| ZymoPURE Plasmid Maxiprep | Zymo Research | Cat#D4203 |
| QIAmp DNA Mini kit | QIAGEN | Cat#51306 |
| Invitrogen Fluorescein-EX Protein Labeling Kit | Thermo Fisher Scientific | Cat#F10240 |
| Amaxa 96-well Nucleofector Kit SF | Lonza | Cat#V4SC-2096 |
| Cell Lines |  |  |
| Human: A549 cells | ATCC | Cat#CCL-185; RRID:CVCL_0023 |
| Human: HEK-293T | ATCC | Cat#CRL-3216; RRID:CVCL_0063 |
| Human: Huh7 WNV replicon and replicon-cured cells | This paper, based on replicon from Lo, M.K.; Tilgner, M.; Shi, P.Y. J Virol 2003, 77, 12901-12906. | N/A |
| Human: PH5CH8 CRIPSR IFNAR1^-/-^ | This paper | N/A |
| Human: THP-1 cells | ATCC | Cat#TIB-202; RRID:CVCL_0006 |
| Monkey: Vero cells | World Health Organization | N/A |
| Mouse: Primary MEF cells | This paper | N/A |
| Mouse: D1-4G2-4-15 hybridoma | ATCC | Cat#HB-112; RRID:CVCL_J890 |
| Oligonucleotides |  |  |
| gFIB-F  TTATTGTCCAACTACCTGTGGC | This study | N/A |
| gFIB-R  GACTTCAAAGTAGCAGCGTCTAT | This study | N/A |
| hHSP70A qPCR/F  ACCAAGCAGACGCAGATCTTC | Yoon, et al. J Biol Chem 2011, 286, 1737-1747 | N/A |
| hHSP70A qPCR/R  CGCCCTCGTACACCTGGAT | Yoon, et al. J Biol Chem 2011, 286, 1737-1747 | N/A |
| IFIT1-F  GCGCTGGGTATGCGATCT | This study | N/A |
| IFIT1-R  CAGCCTGCCTTAGGGGAA | This study | N/A |
| IFITM1-F  TACTCCGTGAAGTCTAGGGACAG | Esser-Nobis et al. J Virol 2019, 93. | N/A |
| IFITM1-R  AACAGGATGAATCCAATGGTCA | Esser-Nobis et al. J Virol 2019, 93. | N/A |
| h_IGFBP1-F  TTGGGACGCCATCAGTACCTA | This study | N/A |
| h_IGFBP1-R  TTGGCTAAACTCTCTACGACTCT | This study | N/A |
| hJAK1-F  CTTTGCCCTGTATGACGAGAAC | This study | N/A |
| hJAK1-R  ACCTCATCCGGTAGTGGAGC | This study | N/A |
| hJAK2-F  TCTGGGGAGTATGTTGCAGAA | This study | N/A |
| hJAK2-R  AGACATGGTTGGGTGGATACC | This study | N/A |
| hJAK3-F  CCTGATCGTGGTCCAGAGAG | This study | N/A |
| hJAK3-R  GCAGGGATCTTGTGAAATGTCAT | This study | N/A |
| NotI/FLAG-hJAK1/F  TTTTTTGCGGCCGCGCCGCCATGGACTACAAGGATGACGATGACAAAATGCAGTATCTAAATATAAAAGAGG | This study | N/A |
| NheI/FLAG-hJAK1/R  TTTTTTGCTAGCTTATTTTAAAAGTGCTTCAAATCCTTC | This study | N/A |
| RPL13A-F  GCCCTACGACAAGAAAAAGCG | Esser-Nobis et al. J Virol 2019, 93. | N/A |
| RPL13A-R  TACTTCCAGCCAACCTCGTGA | Esser-Nobis et al. J Virol 2019, 93. | N/A |
| hTYK2-F  GGAGGAGGGTTCTAGTGGCA | This study | N/A |
| hTYK2-R  ATGTCCCGGAAGTCACAGAAG | This study | N/A |
| WNV-TX EcoRI-(C) s  GGGAATTCGCCATGTCTAAGAAACCAGGAGG | This study | N/A |
| WNV-TX (C)-KpnI as  CCGGTACCCCTCTTTTCTTTTGTTTTGAGC | This study | N/A |
| WNV-TX EcoRI-(NS1) s  GGGAATTCATGATAGCTCTCACGTTTCTCGCAG | This study | N/A |
| WNV-TX (NS1)-KpnI as  ACGGTACCCCAGCATTCACTTGTGACTGCA | This study | N/A |
| WNV-TX SacI-(NS2a) s  CCGAGCTCATGTATAATGCTGATATGATTGA | This study | N/A |
| WNV-TX (NS2a)-KpnI as  ATGGTACCCCGCGTTTACGGTTGG | This study | N/A |
| WNV-TX EcoRI-(NS2b) s  AAGAATTCATGGGATGGCCCGCAACTG | This study | N/A |
| WNV-TX (NS2b)-KpnI as  CCGGTACCCCTCTCTTTGTGTATTGGAGAG | This study | N/A |
| WNV-TX EcoRI-(NS3) s  GAGAATTCATGGGAGGCGTGTTGTGGGACAC | This study | N/A |
| WNV-TX (NS3)-NsiI as  ACCATGCATCCACGTTTTCCCGAGGCG | This study | N/A |
| WNV-TX EcoRI-(NS4a) s  CCGAATTCATGTCTCAGATAGGGCTCATTGA | This study | N/A |
| WNV-TX (NS4a)-KpnI as  ATGGTACCCCCTTCTCTGGCTCAGGAATTA | This study | N/A |
| WNV-TX NsiI-(NS5) s  ATAATGCATATGGGTGGGGCAAAAGGACGCAC | This study | N/A |
| WNV-TX (NS5)-NsiI as  CCCATGCATCCCAATACTGTGTCCTCAACCA | This study | N/A |
| pCAGGS-COOH-TAG seq(f)  GGCAGGGCGGGGTTCG | This study | N/A |
| pCAGGS-HA seq(r)  GCCAGAAGTCAGATGCTCAAG | This study | N/A |
| Wnrep NS5 qPCR/F  GAGTCCAAGAAGTCAGAGGGTACA | Crook et al. Virology 2014, 458-459, 172-182. | N/A |
| Wnrep NS5 qPCR/R  CCACTCTTCATGGTGACAATGTTCC | Crook et al. Virology 2014, 458-459, 172-182. | N/A |
| Recombinant DNA |  |  |
| JAK1 cDNA ORF Clone in Cloning Vector, Human | Sino Biological | Cat#HG11531-U |
| pCAGGS-HA | This study | N/A |
| pCAGGS-WNV-TX(C)-HA | This study | N/A |
| pCAGGS-WNV-TX(NS1)-HA | This study | N/A |
| pCAGGS-WNV-TX(NS2A)-HA | This study | N/A |
| pCAGGS-WNV-TX(NS2B)-HA | This study | N/A |
| pCAGGS-WNV-TX(NS2B3)-HA | This study | N/A |
| pCAGGS-WNV-TX(NS3)-HA | This study | N/A |
| pCAGGS-WNV-TX(NS4A)-HA | This study | N/A |
| pCAGGS-WNV(NS4B)-HA | Adolfo García-Sastre | N/A |
| pCAGGS-WNV-TX(NS5)-HA | This study | N/A |
| pcDNA3.1(+) | Thermo Fisher Scientific | Cat#V79020 |
| pcDNA3.1-ZIKV(C)-FLAG | Tom C. Hobman | N/A |
| pcDNA3.1-ZIKV(NS5)-FLAG | Tom C. Hobman | N/A |
| pRRL-MND-IFNAR1-2A-Puro | This study | N/A |
| pTwist-CMV | This study | N/A |
| pTwist-CMV-HSP90-HA | This study. Custom ordered from Twist Bioscience | N/A |
| pTwist-CMV-FLAG-JAK1 | This study | N/A |
| Software and Algorithms |  |  |
| GraphPad Prism version 8.1.0.325 | GraphPad | RRID:SCR_002798 |
| ImageJ 1.50i | NIH | RRID:SCR_003070 |
| Adobe Photoshop CS5 | Adobe | RRID:SCR_014199 |
| NIS-Elements imaging system software version 4.51 | Nikon Instruments | RRID:SCR_014329 |
| Image Lab version 5.2.1 | Bio-Rad | RRID:SCR_014210 |
| Image Studio Ver 4.0 | LI-COR | RRID:SCR_015795 |
