# Supplementary material for: Flavivirus Nonstructural Protein NS5 Dysregulates HSP90 to Broadly Inhibit JAK/STAT Signaling
Source: Cells. 2020 Apr 7;9(4):899. doi: 10.3390/cells9040899 (PMC7226784; doi:10.3390/cells9040899)
Supplement: Supplementary file 1 [file cells-09-00899-s001.zip › Roby et al - Cells - Supplemental - 28Feb2020.pdf]

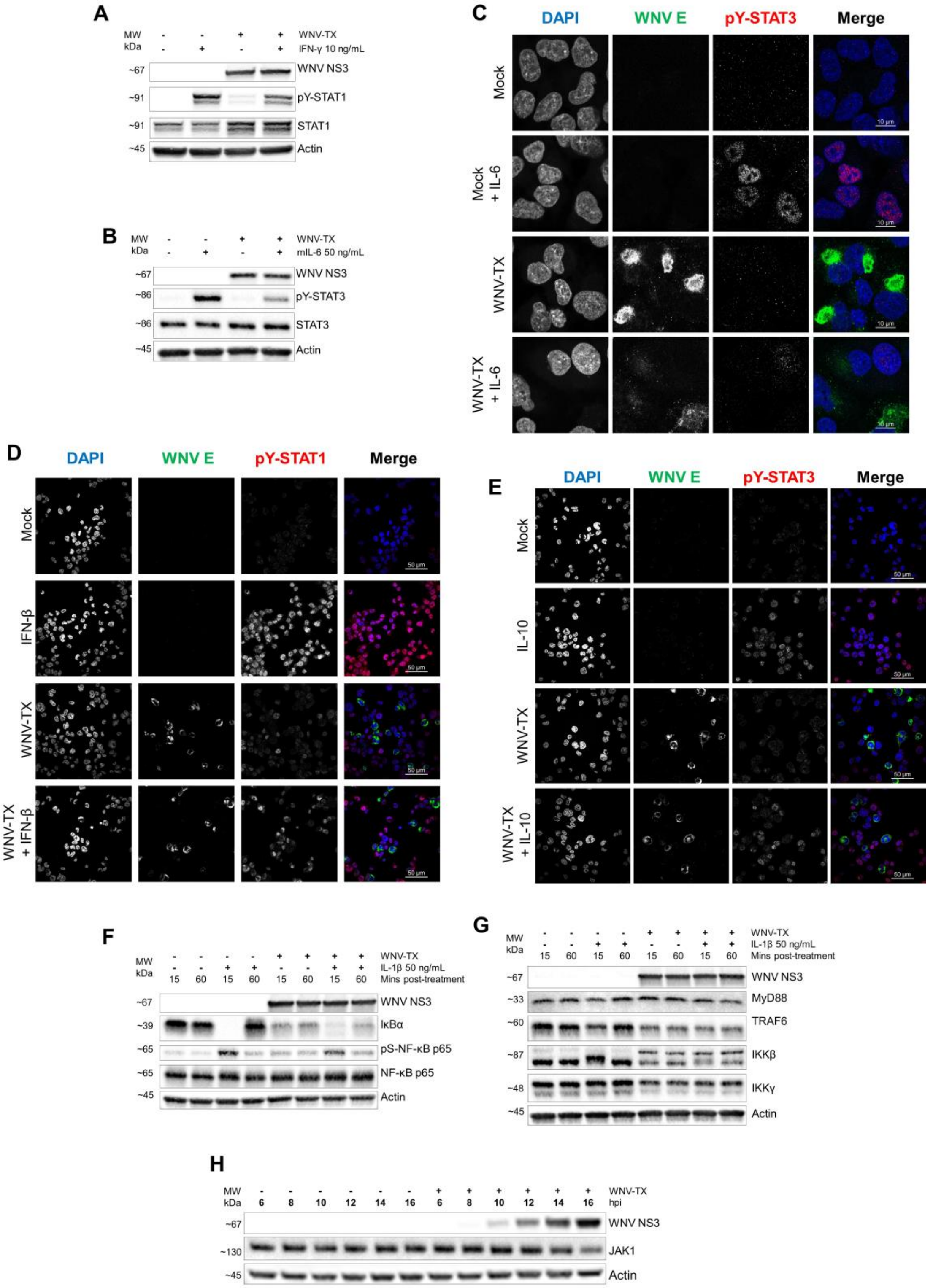

Figure S1: WNV infection inhibits STAT phosphorylation in response to multiple cytokines.
(A) WNV infection of A549 cells attenuates the pY-STAT1 response to IFNγ. (B) WNV infected MEF cells showed attenuated pY-STAT3 to 30 min murine IL6 treatment. (C) A549 cells infected with MOI = 5 WNV for 24 hrs and subsequently treated with 200 ng/mL IL6 for 30 min. WNV infection prevents the accumulation of pY-STAT3 in response to IL6 (arrow heads). THP-1 cells differentiated into macrophages with PMA and infected with MOI = 30 WNV for 24 hrs were unable to generate a pY-STAT1 response to 30 min treatment with (D) 1,000 IU IFNβ, or pY-STAT3 response to 30 min treatment with (E) IL10 (arrow heads). (F) Analysis of downstream signaling in response to IL1β in WNV infected A549 cells. WNV infection leads to reduced abundance of IκBα, however the degree of pS-p65 was largely unaffected by 15 min post-treatment. (G) Proximal signaling components of the IL1β response are altered in WNV infected A549 cells. The abundance of TRAF6, IKKβ, and IKKγ all appear reduced by 24 hpi with WNV. (H) Timecourse experiments revealed that JAK1 abundance starts to diminish between 14 and 16 hpi with MOI = 5 WNV, commensurate with increasing viral protein production. All infections were conducted at MOI = 5 unless otherwise stated. All data are representative of 3 independent experiments or are a combination of all 3 experiments (mean ± SEM). The red pY-STAT3 channel for microscopy images in panels C and E was enhanced equally across all samples to aid visual clarity post-acquisition.

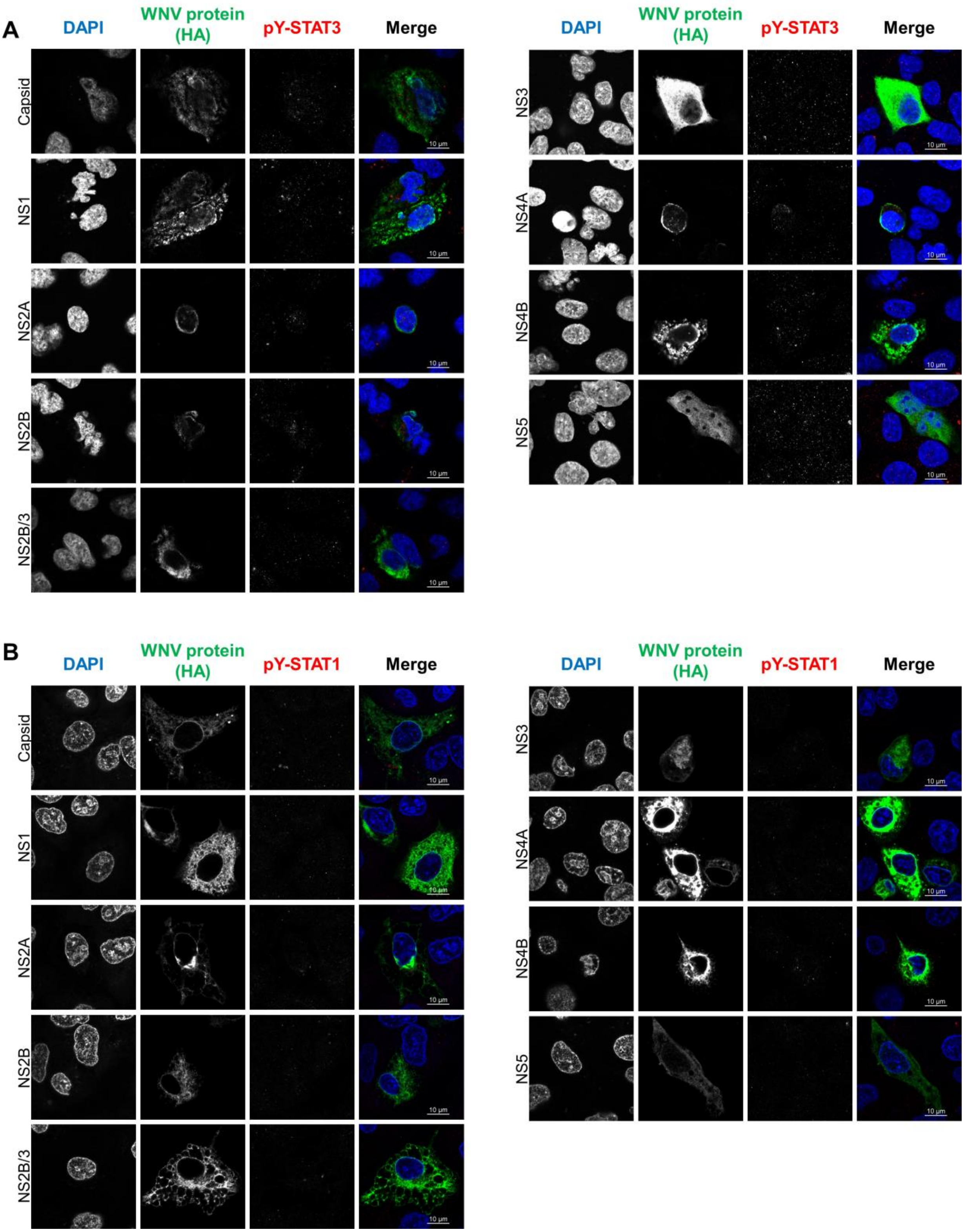

Figure S2: Recombinant WNV protein expression does not induce pY-STAT3 or pY-STAT1.
Expression of recombinant WNV proteins in transfected A549 cells do not by themselves stimulate (A) pY-STAT3 or (B) pY-STAT1. All data are representative of 3 independent experiments. The red pY-STAT3 channel for microscopy images in panel A was enhanced equally across all samples to aid visual clarity post-acquisition.

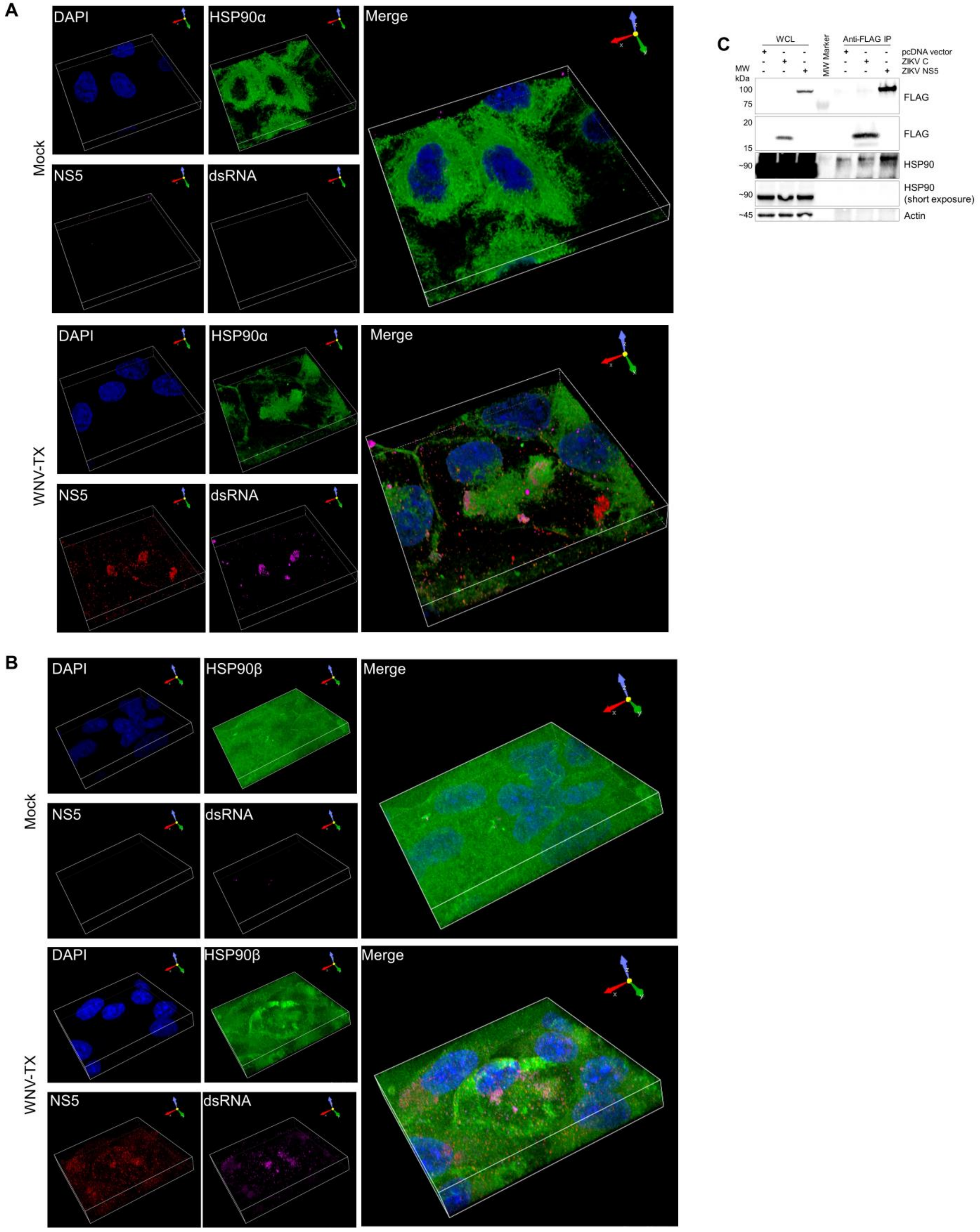

Figure S3: HSP90 interacts with NS5 and is localized to sites of viral dsRNA.
3D rendering reveals discrete co-localization of (A) HSP90α and (B) HSP90β with WNV NS5 and dsRNA in infected A549 cells (arrow heads). The extent of HSP90α/β perinuclear condensation is also revealed in these 3D renderings. (C) Expression of FLAG-tagged ZIKV C and NS5 in HEK-293T cells followed by anti-FLAG co-IP showed endogenous HSP90 interacts specifically with ZIKV NS5. All infections were conducted at MOI = 5 unless otherwise stated. All data are representative of 3 independent experiments.

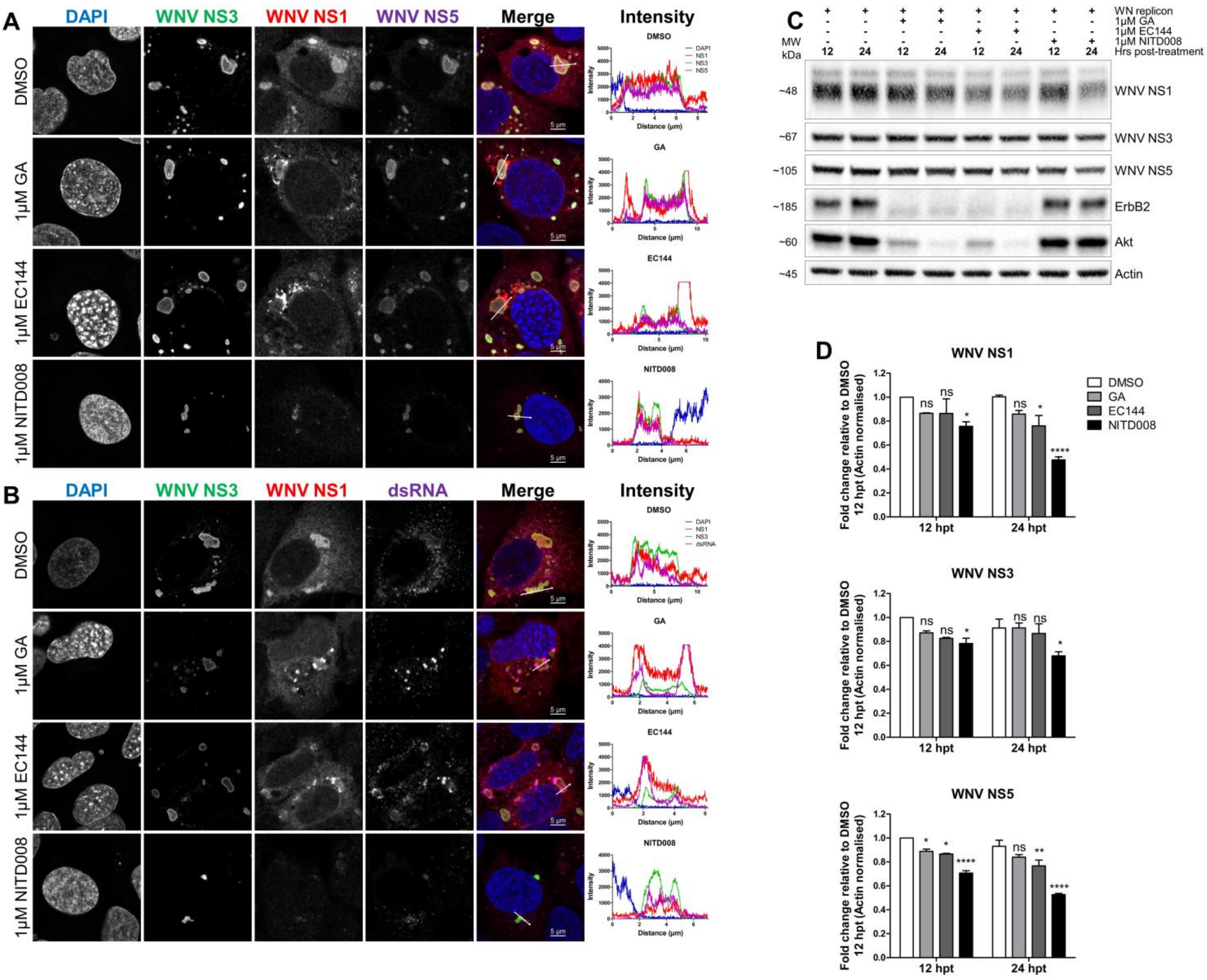

Figure S4: HSP90 inhibition does not perturb WNV replication.

(A-B) Huh7 WNV replicon cells were treated for 24 hrs with DMSO, HSP90 inhibitors GA and EC144, or replication inhibitor NITD008, and stained for NS1, NS3, and either (A) NS5 or (B) dsRNA. HSP90 inhibitors did not alter NS3 and NS5 co-localization, and NS1 moved to bright foci adjacent to NS3/NS5 regions in close association with dsRNA staining (arrow heads). NITD008 did not alter localization, but reduced abundance of NS proteins and RNA. (C, D) Huh7 WNV replicon cells were treated with inhibitors for 12 and 24 hrs and protein stability was assessed. (C) HSP90 inhibitors reduced abundance of Akt and ErbB2, but did not overtly affect amounts of replicon proteins. (D) Quantification of band intensity demonstrated that only NS1 and NS5 displayed slight but significantly reduced abundance upon HSP90 inhibition, in contrast to the notable and significant loss of abundance of NS1, NS3, and NS5 following NITD008 treatment. **** = P < 0.0001, ** = P < 0.01, * = P < 0.05, ns = P>0.05. Two-way repeated measures ANOVA with Holm-Sidak's posttests (D).
